# KidneyTox_v1.0 enables explainable artificial intelligence prediction of nephrotoxicity in small molecules

**DOI:** 10.1038/s41598-026-35496-4

**Published:** 2026-01-13

**Authors:** Sk Abdul Amin, Supratik Kar, Stefano Piotto

**Affiliations:** 1 Department of Pharmacy, University of Salerno, Via Giovanni Paolo II 132, Fisciano, 84084 SA Italy; 2 Chemometrics and Molecular Modeling Laboratory, Department of Chemistry & Physics, Kean University, 1000 Morris Avenue, Union, NJ 07083 USA

**Keywords:** Nephrotoxicity, Chemical space, Fingerprint, Machine learning, qRASAR, Computational biology and bioinformatics, Drug discovery, Nephrology

## Abstract

Drug-induced nephrotoxicity remains a leading cause of kidney dysfunction, often with severe or even fatal outcomes. Computational approaches, in particular artificial intelligence (AI), offer a promising alternative by providing reliable, cost-effective, and ethically sound tools for assessing drug-induced nephrotoxicity. Thereby, potentially reducing reliance on animal testing. This study was driven by three core objectives: (i) to analyze the chemical space of compounds associated with drug-induced nephrotoxicity, (ii) to construct a robust supervised machine learning (ML) model for classification, followed by a quantitative Read-Across Structure-Activity Relationship (qRASAR) study, and (iii) to develop an open-access, eXplainable AI (XAI) platform named “KidneyTox_v1.0” (https://kidneytoxv1.streamlit.app/) for nephrotoxicity prediction. Beyond providing predictions, “KidneyTox_v1.0” offers interpretability through interactive SHAP-based waterfall plots, enabling both domain experts and non-experts to understand the contribution of molecular descriptors to toxicity outcomes. These modelling analyses will assist chemists in designing less nephrotoxic molecules in the future.

**Supplementary Information:**

The online version contains supplementary material available at 10.1038/s41598-026-35496-4.

## Introduction

Kidney toxicity or nephrotoxicity represents a significant bottleneck in the early-stage drug discovery and development, contributing substantively to preclinical and clinical failures and post-market withdrawals^1,2^. The intricate physiological role of kidney in filtering waste products, reabsorbing essential substances, and maintaining homeostasis makes it specifically susceptible to damage by various chemical entities and their metabolites^3^. Nephrotoxicity is especially difficult to predict computationally because kidneys face high drug and metabolite exposure during filtration, with toxicity driven by diverse mechanisms like tubular injury, glomerular damage, oxidative stress, and mitochondrial dysfunction, unlike hepatotoxicity or cardiotoxicity, which often involve more defined pathways. Damage to renal tissue can lead to acute kidney injury (AKI) or progress to chronic kidney disease (CKD), conditions that are associated with significant patient morbidity and mortality and that result in high attrition rates during drug development. Detecting and mitigating probable nephrotoxicity early in the drug discovery pipeline is paramount to minimizing attrition rates, reducing development costs, and ensuring patient safety^4^. Traditional methods for evaluating kidney toxicity, including in vitro cell assays and in vivo animal models, are often time-consuming, resource-intensive, and may not always accurately predict human response^5^. Consequently, there is a critical need for more efficient and predictive approaches to assess the nephrotoxic potential of novel drug candidates.

Now-a-days, the integration of computational approaches, including Machine Learning (ML), Artificial Intelligence (AI), Quantitative Structure-Activity Relationships (QSAR), Read-Across (RA), and quantitative Read-Across Structure-Activity Relationships (qRASAR), have emerged as a powerful paradigm to address the CKD challenges^6,7^. These approaches support the ever-increasing availability of structural and toxicity data to build predictive models that can elucidate the complex interactions between chemicals (drugs) and nephrotoxicity. By analyzing structural scaffolds, molecular fingerprints, along with other physicochemical features, computational tools can assist in identifying structural alerts and key features associated with kidney toxicity, guiding the design of safer drugs with a lower attrition rate^8,9^.

Numerous studies by different research groups have successfully employed AI/ML, and QSAR techniques for predicting various toxicological endpoints, including kidney toxicity^10–12^. Early quantitative approaches showed that ML models, when trained on curated chemical datasets, can capture complex linear as well as nonlinear relationships between molecular fingerprints and toxicity endpoints. For example, researchers have employed techniques ranging from support vector machines (SVM) to random forests (RF) and gradient boosting (GB) methods, consistently achieving improved predictive performance over traditional empirical models^13^. Moreover, by integrating fragment-based analyses to identify toxicophores, specific molecular substructures that are strongly associated with kidney toxicity, these studies have advanced our understanding of the molecular frameworks underlying nephrotoxicity^14^.

RA approaches, often integrated with QSAR, have been employed to predict the toxicity of a target chemical based on the known toxicity of structurally similar compounds^15^. These efforts have demonstrated the potential of computational methods to provide valuable early insights into potential kidney toxicity, complementing traditional experimental methods. However, challenges remain in developing highly accurate and robust predictive models that are broadly applicable across diverse chemical spaces and different mechanisms of nephrotoxicity. Availability of high-quality, curated datasets that encompass both ‘Toxic’ and ‘Non-toxic’ responses is another challenge. Furthermore, making these predictive tools easily accessible and interpretable for medicinal chemists and toxicologists remains a crucial goal^16^. For many years, the heterogeneity and limited size of such datasets have constrained the development of predictive models.

Addressing these challenges, we have developed a comprehensive computational approach and a user-friendly web-based tool, “KidneyTox_v1.0”, for predicting kidney toxicity. Our study utilized a curated dataset of 565 small molecules with experimentally determined ‘Toxic’ and ‘Non-toxic’ responses related to kidney toxicity. We employed a multi-faceted strategy involving (i) chemical space analysis to understand the distribution and diversity of our dataset, (ii) AI/ML-based QSAR modeling followed by qRASAR to build a robust predictive and interpretative model, (iii) development of an open-access, eXplainable AI (XAI) platform for nephrotoxicity prediction.

While specific details of the ML model employed will be discussed in the methods section, it was selected based on its performance and interpretability. The developed predictive model is integrated into the “KidneyTox_v1.0” (https://github.com/Amincheminform/KidneyTox_v1.0), providing an open-source platform for users to obtain rapid predictions. Users can easily input a chemical structure either by providing its SMILES string or by directly drawing the structure within the intuitive Streamlit-based web interface. Beyond a simple binary prediction, the platform generates insightful visualizations, including applicability domain (AD) analysis, waterfall plots that illustrate the positive and negative contributions of major descriptors/features towards the predicted nephrotoxicity. Furthermore, users receive comparative information on chemicals within the modeled chemical space that are structurally similar to their query chemicals, along with their respective predicted outcomes and accompanying waterfall plots. This comparative analysis helps contextualize the predicted nephrotoxicity of the query molecule within the landscape of known compounds, enhancing the interpretability and functionality of the predictions.

## Results

A dataset of drug-induced nephrotoxicity (*N*_*Total*_ = 565) was sourced from literatures^17,18^. This dataset comprises 565 chemically diverse small molecules, including 287 drugs reported to induce nephrotoxicity in humans (‘Toxic’) and 278 drugs with no known nephrotoxic effects (‘Non-toxic’) as given in Table S1 of the Supplementary material.

### Analysis of the chemical space

**Fig. 1:**
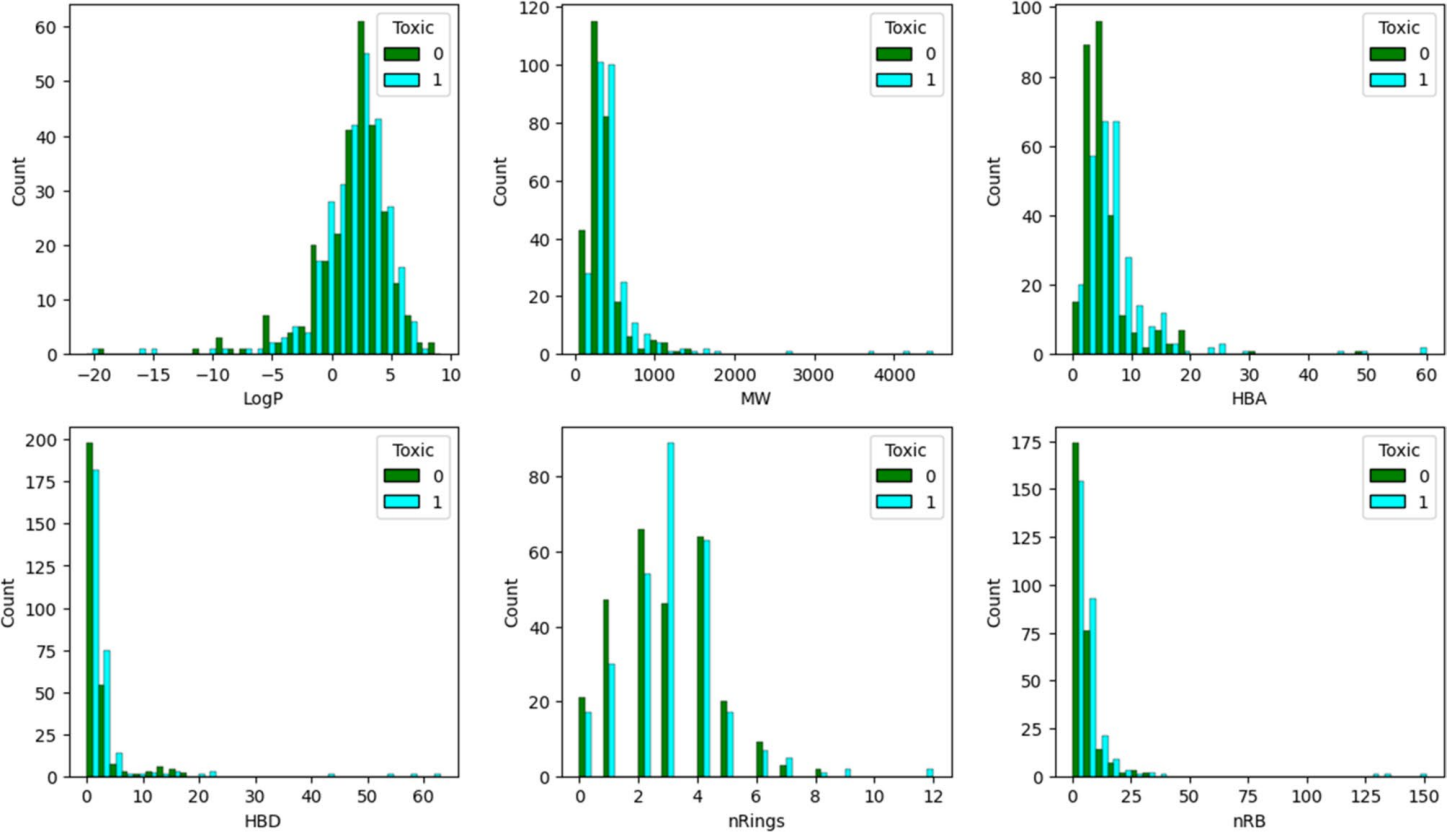
Bin plots of common features such as *LogP*, *MW*, *HBA*, *HBD*, *nRings*, and *nRB*.

Chemical space plays a crucial role in both chemical, biological and toxicological research, particularly in pharmaceutical chemistry. Figure 1 illustrates the frequency distribution of six molecular properties (octanol-water partition coefficient (*LogP*), molecular weight (*MW*), hydrogen bond acceptors (*HBA*), hydrogen bond donors (*HBD*), total number of ring systems (*nRings*), number of rotatable bonds (*nRB*)) of the molecules investigated. The molecule with the highest *LogP* value is highly lipophilic with a value of 9.16 (compound 368). Conversely, compound 479 is the most hydrophilic, with a *LogP* value of -20.60. The average *LogP* value across all molecules in the dataset is 1.81, indicating that most molecules are moderately lipophilic. There are 142 molecules without aromatic rings, 155 molecules with one aromatic ring, 166 molecules with two aromatic rings, and 72 molecules with three aromatic rings. Hence, on average, molecules have one or two aromatic rings. There are 19 molecules with four aromatic rings, 8 molecules with five aromatic rings, 2 molecules (example: compounds 396 and 483) with six aromatic rings, and 1 molecule (example: compound 176) with nine aromatic rings. Moreover, compound 176 also exhibits 151 rotatable bonds. Molecules across the datasets typically have around 7 rotatable bonds, indicating moderate flexibility.

There are 31 molecules without any rotatable bond and 38 molecules with one rotatable bond. On average, molecules have about 3 rings. The average *HBA* and *HBD* values across all dataset molecules are 6.10 and 2.89, respectively. Hence, most molecules have around 6 hydrogen bond acceptors and around 3 hydrogen bond donors. The average topological polar surface area (*TPSA*) and *MW* values across all dataset molecules are 113.53 and 416.87, respectively. Compound 176 (*MW* = 4491.95) is the largest molecule by weight, whereas compound 119 (*MW* = 59.04) is the smallest molecule by weight. The average *MW* (416.87) of molecules indicates that most are small to medium-sized. The broad ranges of molecular properties, particularly *LogP*, *MW*, and *TPSA*, suggest a diverse chemical space, including small organic compounds, and highly flexible structures.

### Fingerprint-assisted scaffold diversity analysis

**Fig. 2:**
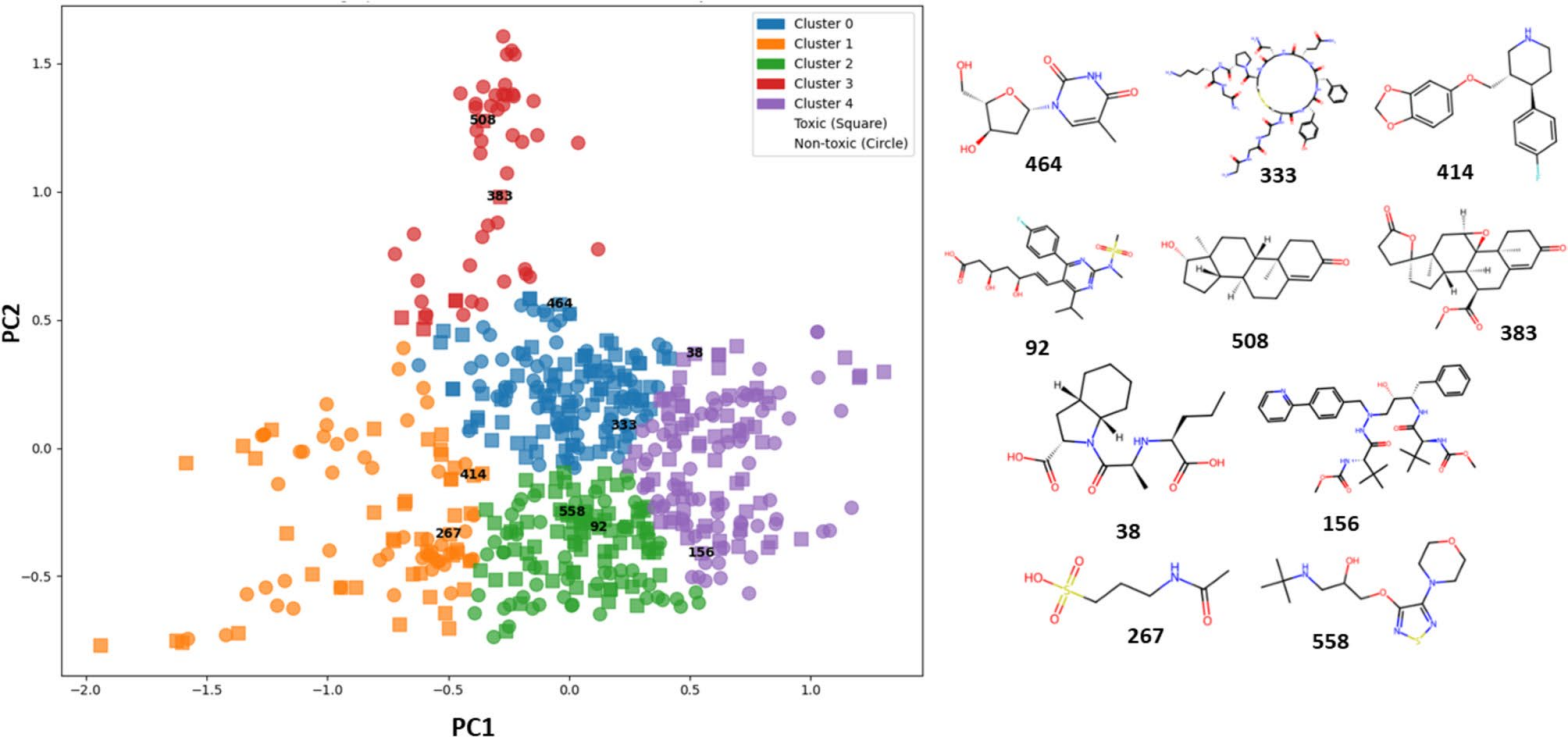
The PCA scatter plot highlights two representative drugs [Perindoprilat (compound 38), Rosuvastatin (compound 92), Atazanavir (compound 156), Acamprosate (compound 267), Terlipressin (compound 333), Eplerenone (compound 383), Paroxetine (compound 414), Telbivudine (compound 464), Testosterone (compound 508), Timolol (compound 558)] from each cluster.

The fingerprint-assisted scaffold diversity analysis (integrating cheminformatics and unsupervised ML methods) was performed to gain insights into the scaffold diversity^19^. Here, we used the open-access tool “Fasda_v1.0” (https://github.com/Amincheminfom/Fasda_v1) to perform fingerprint-assisted scaffold diversity analysis of compounds associated with drug-induced nephrotoxicity. First, *ECFP_6* fingerprints were generated, and pairwise similarities were computed using the Tanimoto coefficient. Principal Component Analysis (PCA) was applied for 2D visualization, followed by *K*-Means clustering (Elbow plot for determining the optimal number of clusters has been provided in Figure S1 of the Supplementary material) to partition the molecules into five chemically similar groups. Then the analysis of unique and singleton scaffolds highlighted overall structural diversity within each cluster. Two representative drugs [Perindoprilat (compound 38), Rosuvastatin (compound 92), Atazanavir (compound 156), Acamprosate (compound 267), Terlipressin (compound 333), Eplerenone (compound 383), Paroxetine (compound 414), Telbivudine (compound 464), Testosterone (compound 508), Timolol (compound 558)] from each cluster are provided in Fig. 2.

Cluster 0 stands out for having a high number of compounds (M = 149), Bemis-Murcko scaffolds (*N* = 126), and singleton scaffolds (Ns = 108), indicating a high degree of structural diversity (Table 1). The high singleton ratio (0.857) also reinforces that most scaffolds in this cluster occur only once, reflecting high structural diversity. Cluster 1 is expressed by 52 distinct Bemis-Murcko scaffolds with a singleton ratio of 0.827. It can be observed from Table 1 that cluster 2 has 134 compounds, with a singleton ratio of 0.876. Moreover, cluster 3 (M = 50) and cluster 4 (M = 139) exhibit Bemis-Murcko scaffolds of 32 and 99, respectively. The singleton ratio is 71.9% and 89.9% for cluster 3 and 4, respectively. Hence, all the clusters exhibit singleton ratios above 70%, highlighting consistently high structural diversity across the dataset.

**Table 1:** Scaffold content and diversity calculation of the dataset of drug-induced nephrotoxicity.

Cluster	M	*N*	Ns	Singleton Ratio
0	149	126	108	0.857
1	93	52	43	0.827
2	134	97	85	0.876
3	50	32	23	0.719
4	139	99	89	0.899

Number of compounds (M), Number of unique scaffolds (N), Number of singleton scaffolds (Ns), Singleton Ratio is the ratio of singleton scaffolds to total scaffolds.

### Machine learning studies

#### Calculation of the descriptors and data pre-treatment

Mordred descriptors^20^ were calculated by using *in house* “Fiore_v1.0” platform, in particular, *Fiore_FC* (Feature Calculation) tool (https://github.com/Amincheminfom/Fiore_v1.0) prior to developing AI/ML models. Columns containing text information, constant columns (those with identical values across all samples) were deleted to eliminate unnecessary noise and reduce dimensionality^21^. Notably, the dataset was divided into 80% training and 20% test sets using repeated random stratified sampling, iterating over 50 different random splits. These 50 independent train-test splits were used to develop preliminary Random Forest Classifier (RFC) models, allowing the assessment of model robustness rather than relying on a single partition. The best-performing split was selected, and the results are depicted in Figure S2 of the Supplementary material. Subsequently, feature selection was performed using the selected training set, followed by model development with RFC model optimized through Optuna-based hyperparameter tuning (https://optuna.org/).

### Feature selection

**Fig. 3:**
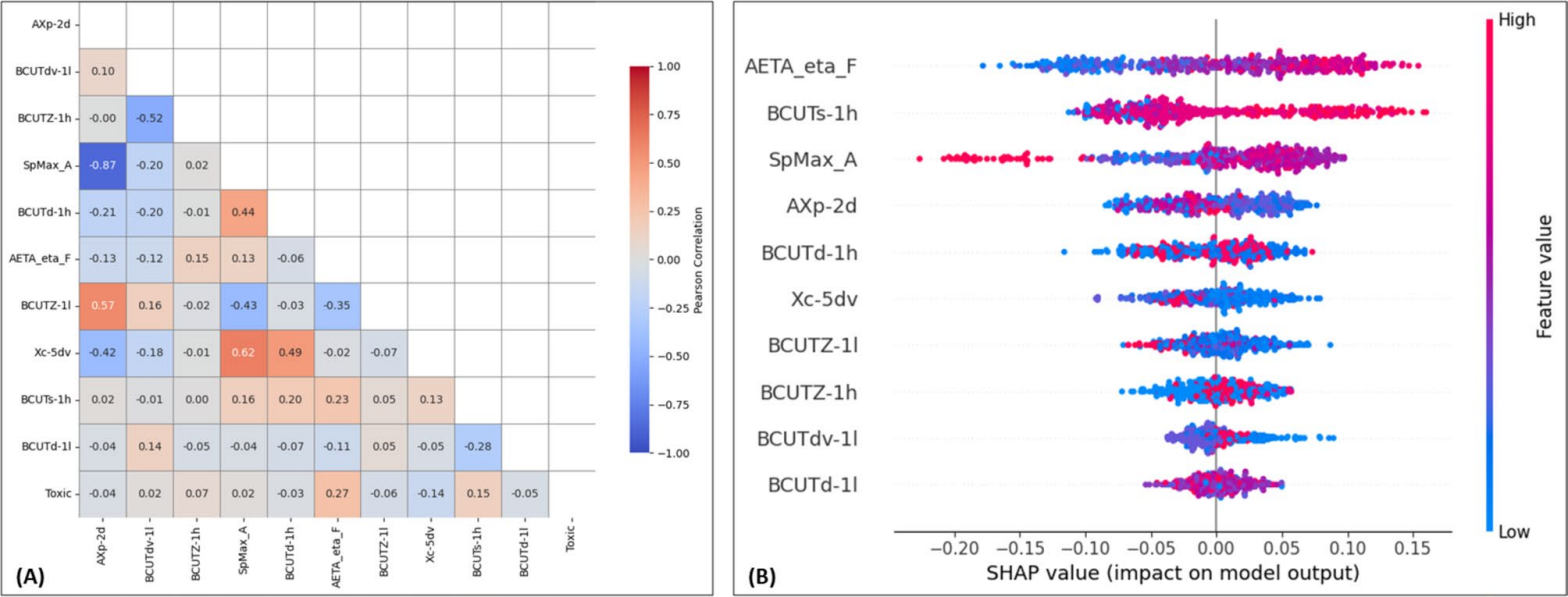
(**A**) Heatmap of the correlation matrix of the selected descriptors. (**B**) Summary plot of SHAP values for each descriptor of the training set.

Feature selection is a critical preprocessing step in ML that enhances model performance^22^. This step reduces complexity and preserves essential information, ensuring model stability during training. Meanwhile, information gain (or mutual information)^23^ was calculated to identify features most relevant to predict the target variable (toxicity value). Finally, features with importance scores greater than 0.05 were selected to improve accuracy and reduce computational costs. The selected descriptors collectively provide a detailed representation of molecular structure, properties, and reactivity, making them powerful tools for ML. A heatmap of the correlation matix of the selected descriptors is depicted in Fig. 3A.

### Machine learning model development

The optimal hyperparameters identified during the tuning process^24^ are as follows: *n_estimators* = 60, *max_depth* = 24, *min_samples_split* (minimum number of samples required to split a node) = 13, *min_samples_leaf* (minimum number of samples that a leaf node must have) = 2. The value of *n_estimators* suggests that the model achieves optimal performance with 60 individual trees. The selected value primarily reflects empirical performance saturation rather than computational constraints. Together, all these hyperparameters (*n_estimators* = 60, *max_depth* = 24, *min_samples_split* = 13, *min_samples_leaf* = 2) produce a model with strong discriminative power while maintaining a reasonable level of complexity. The performance metrics of the training set include an Accuracy of 94.25%, Precision of 94.83%, Recall of 94.02%, and an F1 Score of 94.42%, highlighting the excellent quality of the Optuna-optimized RFC model. Additionally, this model performs well in the validation dataset, achieving a best accuracy score of 0.841, precision of 0.830, recall of 0.830 and F1 score of 0.830 on the test set, indicating its ability to capture generalizable patterns. Furthermore, ten alternative train-test splits were evaluated to develop additional RFC models. These splits yielded moderately lower performance (Table S2 of the Supplementary material) than the original RFC model, with accuracies ranging from 0.673 to 0.796 and corresponding variations in precision, recall, F1-scores, and optimized hyperparameters (e.g., Split 1: Accuracy = 0.673, F1 = 0.704; Split 6: Accuracy = 0.796, F1 = 0.803). These findings indicate that the original RFC model exhibits robust and the best predictive behavior across replicate splits. Finally, the plot of selected descriptors with their importance score as per the final RFC model is given in Figure S3A of the Supplementary material.

### Applicability domain (AD) analysis

The AD^25,26^ is important for assessing the uncertainty in predicting a specific molecule. According to Principle 3 of the Organization for Economic Co-operation and Development (OECD) guidelines^27^, it is essential to define the AD when applying validated models to predict new data points. The predictability of an ML model is considered reliable only if the compound being analyzed falls within the AD. Here, leverage approach is considered to define the X-outliers (training set) and identify the molecules that reside outside the AD (in the case of the test set). In this study, the number of descriptors is 10, the number of training samples is 452, hence the leverage threshold is found to be 0.066. The results suggest that the test set compounds 200 (Leverage: 0.229), 352 (Leverage: 0.101), 171 (Leverage: 0.101), 119 (Leverage: 0.187) are the outliers because the leverage values of these compounds are exceed the threshold value (0.066) as depicted in Figure S3B of the **Supplementary material**.

### Interpretation of the descriptors through SHAP (SHapley additive exPlanations) plot

The RFC model identified different types of descriptors, such as BCUT descriptors, autocorrelation descriptors, topological descriptors, electronegativity and dipole-related descriptors. BCUT descriptors (e.g., *BCUTdv-1 L*, *BCUTZ-1 h*) describe molecular size, shape, and electronic properties, whereas autocorrelation descriptors (e.g., *AXp-2d*, *Xc-5dv*) offer insights into the spatial distribution of properties like charge or electronegativity^28^ (Table 2). Topological descriptors (e.g., *SpMax_A*) capture connectivity and structural features. Meanwhile, electronegativity and dipole-related descriptors (e.g., *AETA_eta_F*) reflect molecular reactivity and interaction potential, crucial for activity predictions.

**Table 2:** A brief explanation of the selected descriptors.

Descriptors	Types	Definition
*BCUTdv-1 L*	BCUT descriptors	A BCUT descriptor derived from eigenvalues of an adjacency matrix representing the molecule, weighted by a specific property (here, van der Waals volume). The “1l” indicates the lowest eigenvalue.
*BCUTZ-1 h*	BCUT descriptors	Similar to BCUTdv-1 L, but the matrix is weighted by atomic polarizability, and “1 h” refers to the highest eigenvalue.
*BCUTd-1 h*	BCUT descriptors	A BCUT descriptor where the adjacency matrix is weighted by a dipole-related property. “1 h” indicates the highest eigenvalue.
*BCUTZ-1 L*	BCUT descriptors	A BCUT descriptor weighted by atomic polarizability, where “1l” refers to the lowest eigenvalue.
*BCUTd-1 L*	BCUT descriptors	A BCUT descriptor weighted by dipole-related properties, focusing on the lowest eigenvalue.
*BCUTs-1 h*	BCUT descriptors	A BCUT descriptor weighted by atomic electronegativity, focusing on the highest eigenvalue.
*AXp-2d*	Autocorrelation descriptors	This is a 2D autocorrelation descriptor that measures the distribution of atomic properties (like electronegativity, mass, or charge) weighted by bond distance in a molecule.
*Xc-5dv*	Autocorrelation descriptors	A 2D autocorrelation descriptor that considers the distribution of valence electron information across a molecule.
*SpMax_A*	Topological descriptors	This descriptor represents the maximum eigenvalue of the adjacency matrix weighted by atomic properties. The “_A” suffix suggests a specific property, such as electronegativity or polarizability.
*AETA_eta_F*	Dipole-related descriptors	A measure of atomic electronegativity weighted topological descriptor. It specifically relates to the F (fluorine) atom or fluorine-related features in the molecule.

**Fig. 4:**
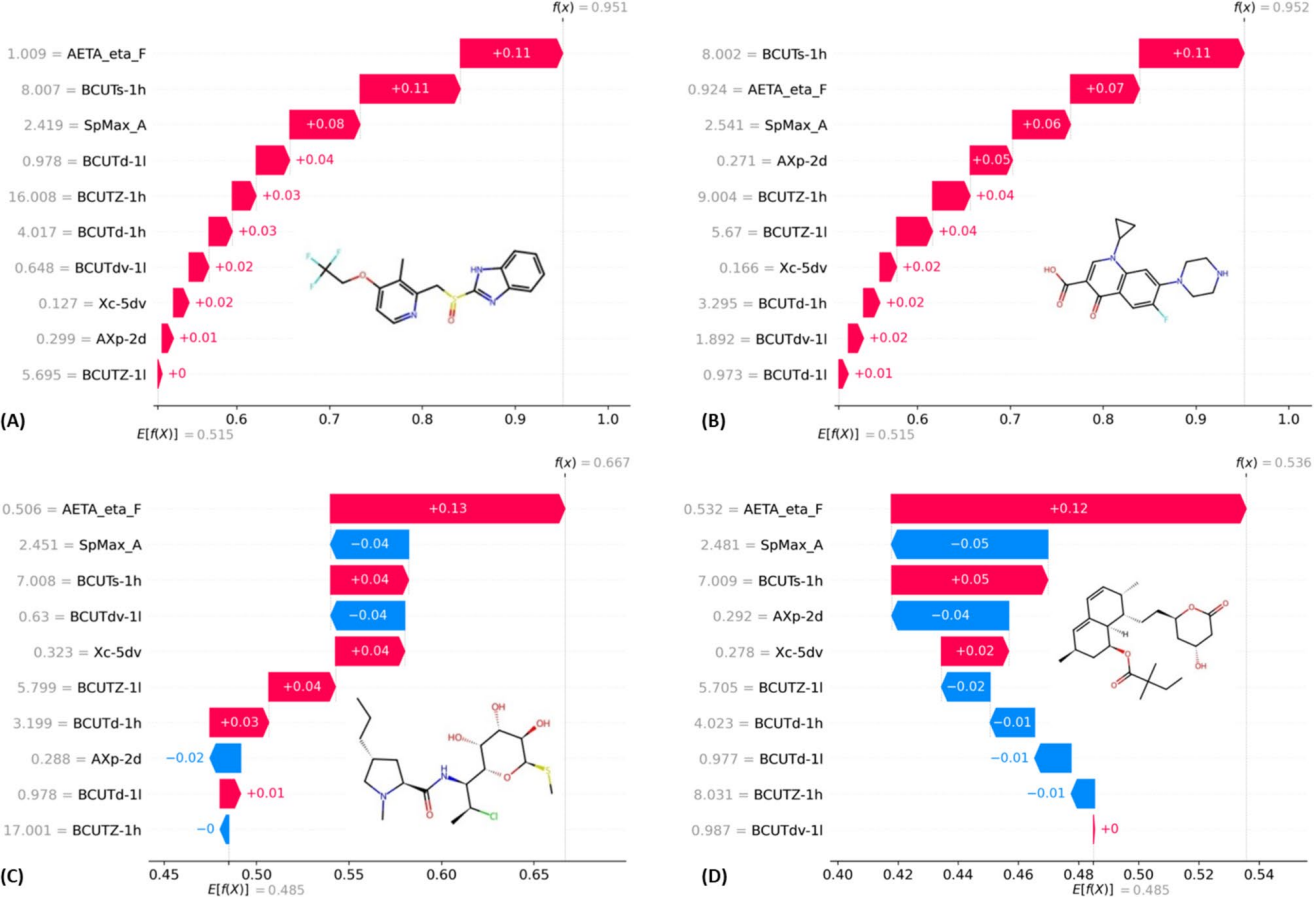
Waterfall plots of the (**A**) Lansoprazole (compound 366), (**B**) Ciprofloxacin (compound 400), (**C**) Clindamycin (compound 79), (**D**) Simvastatin (compound 364).

The SHAP (SHapley Additive exPlanations; https://shap.readthedocs.io/en/latest/) summary plot of the training set descriptors (*AXp-2d*, *BCUTdv-1 L*, *BCUTZ-1 h*, *SpMax_A*, *BCUTd-1 h*, *AETA_eta_F*, *BCUTZ-1 L*, *Xc-5dv*, *BCUTs-1 h*, and *BCUTd-1 L*) is presented in Fig. 3B. In this plot, the color gradient from blue to red represents low to high feature values, respectively. This color-coding helps to interpret the magnitude of each descriptor in the impact of the prediction of the RFC model. For example, the descriptor *AETA_eta_F* displays a broad distribution of both positive and negative SHAP values, indicating that its effect on the model outcome is context-dependent and influenced by interactions with other descriptors. In general, higher values of *BCUTs-1 h* (shown in red) are associated with positive SHAP values, indicating that increased atomic electronegativity in certain molecular regions tends to drive predictions toward kidney toxicity (see Fig. 4). In contrast, for *SpMax_A*, higher feature values (red) are often associated with negative SHAP values, suggesting that molecules with overall higher topological polarizability, such as Clindamycin (compound 79), are more likely to be predicted as ‘Non-toxic’. Additionally, the SHAP waterfall plot (Fig. 4) provides a detailed breakdown of the contribution of individual features to the final prediction for a specific compound.

From the waterfall plot of Fig. 4A, it can be observed that the *AETA_eta_F* provides the largest positive contribution, significantly increasing the prediction toward the ‘Toxic’ class for the investigated molecules. The next important descriptors *BCUTs-1 h*, and *SpMax_A* exhibit good contributions, while the *AXp-2d*, *BCUTZ-1 L* having poor contributions for Lansoprazole (compound 366). This drug is a proton pump inhibitor (PPI) which is used in peptic ulcer disease, Zollinger-Ellison syndrome. It can lead to chronic kidney toxicity. Similarly, from the waterfall plot of Ciprofloxacin (Fig. 4B), it can be noticed that *BCUTs-1 h*,* AETA_eta_F*, and *SpMax_A* provide the largest positive contribution, significantly increasing the prediction toward the ‘Toxic’ class. It is a commonly used antibiotic that has been linked to nephrotoxicity.

On the other hand, *SpMax_A* provides the largest negative contribution, significantly increasing the prediction toward the ‘Non-toxic’ class for Clindamycin (compound 79, Fig. 4C), and Simvastatin (compound 364, Fig. 4D). Collectively, these observations indicate that molecular characteristics such as size, shape, electronic properties, aromaticity, and the spatial distribution of features like charge or electronegativity play a critical role in nephrotoxicity. These can be mechanistically linked to kidney-specific toxicity. For example, polarity and dipole moment may influence renal tubular accumulation and secretion, while aromaticity may promote bioactivation to reactive metabolites that induce oxidative stress or mitochondrial damage in kidney cells. However, kidney toxicity is not solely determined by these molecular attributes; it is also influenced by external factors including dosage, duration of exposure, individual patient-specific variables (e.g., genetic predisposition, pre-existing renal conditions), and potential drug-drug interactions.

### Descriptor insights and interpretability of qRASAR study

In this study, qRASAR classification models were developed using descriptors derived from similarity and error-based metrics under three kernel functions: Euclidean (EUC), Gaussian (GK), and Laplacian (LK)^29^. Each model incorporated 10 ML-derived descriptors along with 8 RASAR descriptors per kernel. Feature selection was performed based on important rankings to derive simplified versions of each model, enhancing interpretability without significantly compromising performance. Feature importance rankings prompted the development of reduced models, preserving only the top three descriptors, typically MaxSim, MaxDist, and the Banerjee-Roy coefficient. Table S3 of the Supplementary material highlighted the detailed description of RASAR descriptors. We have developed 6 classification-based qRASAR models where three are combining ML-derived features and error-based features, while 3 models are developed based on only error-based features. Complete results are summarized in Table S4 of the Supplementary material. Among all selected (reduced) models, the EUC (Selected) model outperformed its GK and LK counterparts, achieving a test accuracy of 0.7568, precision of 0.7400, recall of 0.7255, and an F1-score of 0.7327. This indicates a strong balance between sensitivity and specificity and establishes EUC (Selected) as the best-performing model, despite using only three error-based descriptors. When descriptors were reduced to the top three features in the selected models, there was a slight drop in performance across all kernels. For instance, the LK-selected model exhibited an F1-score of 0.6535 compared to 0.7475 in the full model. This drop highlights the contribution of additional descriptors to model robustness, even though the selected models maintained reasonable accuracy.

Considering the number of features and classification result, EUC (Selected) has been selected as the best model among all six developed models. Feature importance analysis consistently identified three key descriptors across all kernels. The three descriptors in the EUC (Selected) model were:

- *MaxWtSim(EUC)*: The maximum weighted similarity to analogs, incorporating both chemical similarity and the weight of experimental activity.
- *MaxNeg(EUC)*: The maximum similarity to inactive analogs, acting as a countermeasure to false positives by identifying structural proximity to known inactives.
- *gm(EUC) (Banerjee–Roy coefficient)*: A geometric mean of local error contributions, quantifying inconsistency between predicted and observed activities in the chemical neighborhood.

These descriptors are not arbitrary. They are error-based representations derived from ML-selected molecular features, meaning the structural information originally captured by the ML model is now encoded into interpretable, chemically meaningful descriptors^30^. This transformation enables the model to retain predictive strength while offering transparency, an essential requirement for regulatory applications and read-across justifications. The ability of a model with just three error-based descriptors to outperform more complex models illustrates the power of feature distillation through similarity-weighted and error-penalized transformations. These descriptors reflect both the confidence and consistency of predictions based on analogs and are rooted in established chemical read-across principles. The qRASAR framework provides an advantage over standard QSAR by integrating similarity-based reasoning, which allows local mechanistic insights into why structurally related compounds share nephrotoxic outcomes. In contrast to black-box deep learning, which may offer high accuracy but limited transparency, qRASAR predictions are chemically interpretable and directly actionable, linking descriptors and scaffolds to toxicity mechanisms.

### “KidneyTox_v1.0”: an open-access XAI platform for predicting nephrotoxicity

**Fig. 5:**
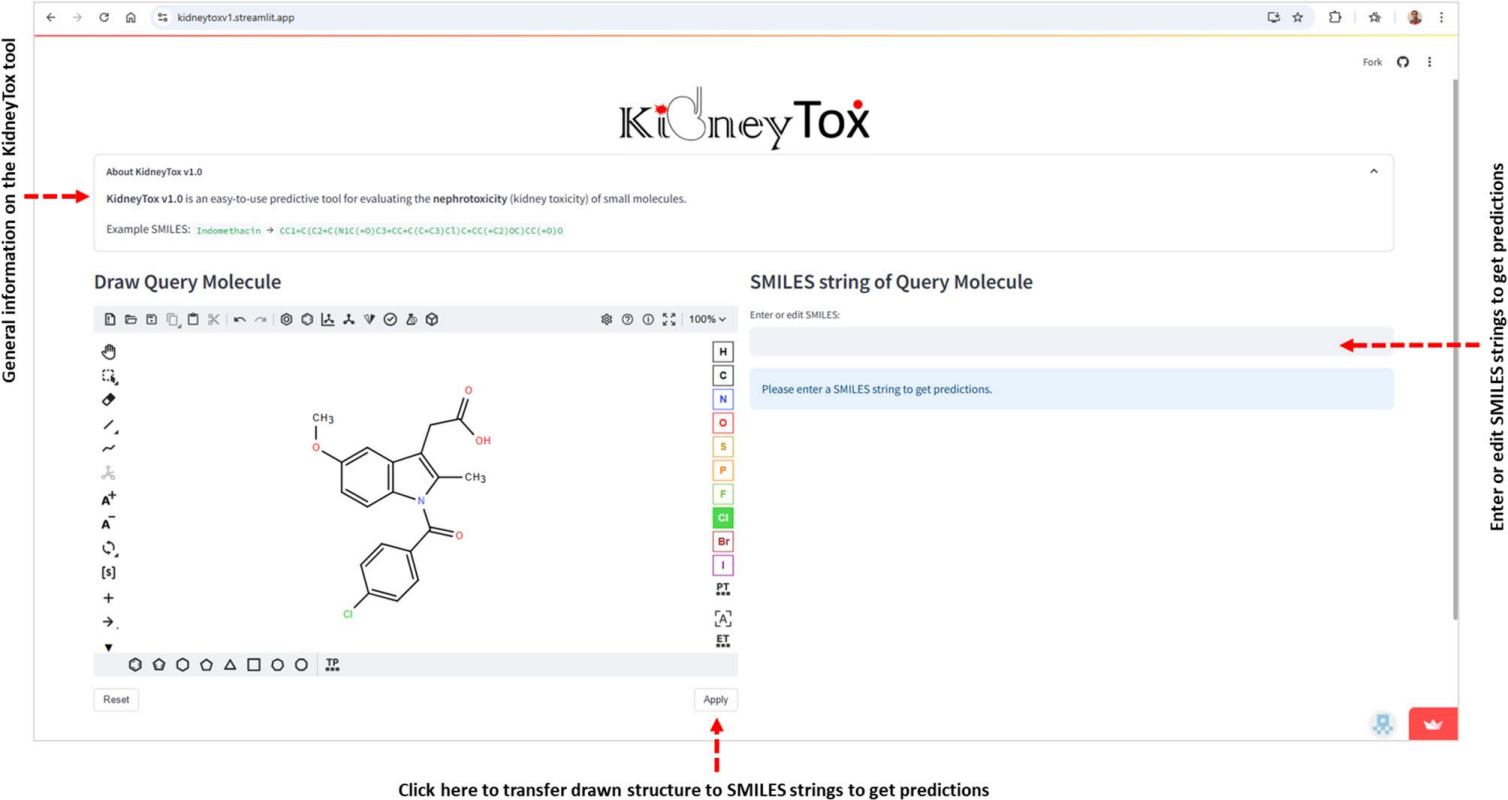
The interface of “KidneyTox_v1.0” tool. The actual input is a SMILES string. Query molecules can be directly pasted or typed in SMILES format or inserted through the molecular sketcher. If user directly pasted or typed or Edited in SMILES strings, then the calculations/predictions can be started by pressing “Enter”. If the user uses the molecular sketcher, then the predictions can be started by clicking on the “Apply” button.

**Fig. 6:**
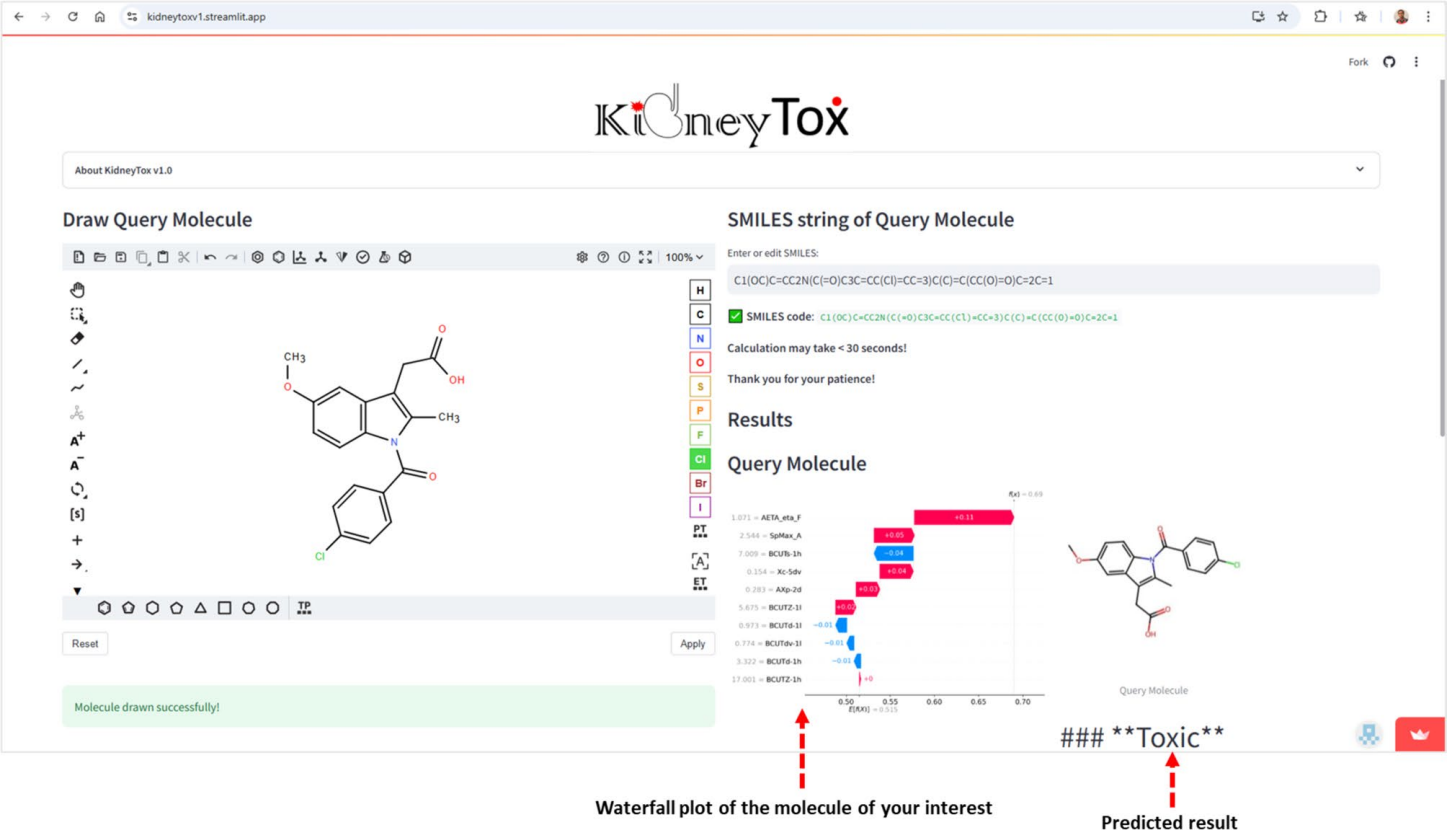
The visualization of 2D structures, predicted results (either ‘Toxic’ or ‘Non-toxic’), applicability domain analysis results, and the SHAP waterfall plots of the query molecule, and the most similar molecule from the dataset with respect to the query molecule. The panel is headed by the query molecule and an up-arrow button to scroll to the top of the page. Users can download the plot(s) and the 2D structure(s).

This Streamlit (https://streamlit.io)-based web application provides a versatile and robust framework for predicting the potential risk of nephrotoxicity associated with small molecules (Fig. 5). Based on a rigorously validated AI/ML model, the “KidneyTox_v1.0” (https://kidneytoxv1.streamlit.app/) classifies query compounds into two clear categories: ‘Toxic’ or ‘Non-toxic’. This platform predicts the outcomes for external data (query molecules) using our pre-trained ML model and provides a detailed explanation of the predictions using SHAP analysis (Fig. 6). Users can conveniently input the SMILES string or directly draw the chemical structure of the query molecule within an intuitive, Python-based web application interface. Users also receive waterfall plots to contextualize the predicted nephrotoxicity of their query molecules. In addition, this platform offers the visualization of the most similar molecule from the dataset concerning the query molecule (based on the Tanimoto-similarity). The employed dataset and codes are available on GitHub (https://github.com/Amincheminform/KidneyTox_v1.0).

In Fig. 6, an example prediction *via* “KidneyTox_v1.0” tool is depicted. It offers the visualization of 2D structures, predicted results (either ‘Toxic’ or ‘Non-toxic’), and the SHAP waterfall plot of the query molecule, the most similar molecule from the dataset to the query molecule. Importantly, this user-friendly XAI platform serves two primary purposes:

- *Facilitating accessibility and knowledge dissemination*: By visualizing 2D molecular structures alongside SHAP waterfall plots for both query compounds and known kidney toxic references, the “KidneyTox_v1.0” platform provides interpretable insights into feature contributions toward nephrotoxicity predictions. Meanwhile, for each query molecule, this tool calculates the leverage value based on the descriptors relative to the training set. Molecules with leverage below the defined threshold are considered “Within AD”, indicating reliable predictions, while molecules above the threshold are “Outside AD”, signalling caution. This information is displayed alongside the prediction, with “Within AD” shown in green and “Outside AD” in red. This enhanced interpretability supports more informed decision-making.
- *Enabling hypothesis generation for future research*: This platform also influences the rational design of novel ‘Non-toxic’ chemical scaffolds by providing insights from descriptor contributions. This capability is particularly valuable for advancing drug discovery efforts in nephrotoxicity prediction, especially in contexts lacking large-scale screening data.

## Discussion & conclusion

This study presents an integrative cheminformatics and AI/ML framework for predicting drug-induced nephrotoxicity using a chemically diverse dataset of 565 compounds. The dataset exhibits diverse chemical space, with most molecules being moderately lipophilic (average *LogP* = 1.81), containing one or two aromatic rings, ~ 7 rotatable bonds, ~ 6 hydrogen bond acceptors, and ~ 3 hydrogen bond donors. Average *TPSA* and *MW* are 113.5 Å^2^ and 416.9, respectively, with extremes ranging from highly hydrophilic to highly lipophilic compounds. Hence, comprehensive chemical space analysis revealed a broad distribution of molecular properties, underscoring the structural and physicochemical diversity of nephrotoxic and non-toxic drugs. Moreover, scaffold diversity analysis using “Fasda_v1.0” confirmed high uniqueness across five clusters. Cluster 0 shows the highest diversity with 149 compounds, 126 Bemis-Murcko scaffolds, and a singleton ratio of 0.857. Clusters 1–4 also display high diversity, with singleton ratios ranging from 71.9% to 89.9%. Overall, all clusters carry more than 70% singleton scaffolds, underscoring the high structural diversity across the dataset.

We developed an Optuna-optimized RFC model (best parameters: *n_estimators* = 60, *max_depth* = 24, *min_samples_split* = 13, *min_samples_leaf* = 2) using selected Mordred descriptors, achieving excellent performance (Accuracy = 0.841, Precision = 0.830, Recall = 0.830, F1 = 0.830). SHAP-based XAI analysis identified key descriptors (e.g., *AETA_eta_F*, *BCUTs-1 h*, *SpMax_A*) as significant contributors to toxicity prediction, with interpretable trends linking electronegativity, flexibility, and polarizability to nephrotoxicity outcomes. Notably, waterfall plots illustrated descriptor-specific impacts on known nephrotoxic (e.g., Lansoprazole, Ciprofloxacin) and non-toxic (e.g., Simvastatin) drugs, confirming model interpretability. Meanwhile, the identified outliers such as compounds 119 (a very small and highly polar carboxylate), 171 (a compact N–C–C–S fragment with atypical electronegativity patterns), 200 (an N-rich polycyclic aromatic system with high conjugation and polarizability), and 352 (a large and structurally complex peptidic boronic-acid scaffold) deviate markedly from the physicochemical space of the dataset. Consistent with this, these outliers (compounds 119, 171, 200, and 352) exhibit extreme values in key descriptors (Table S5 of the Supplementary material). For example, unusually low or high *BCUT* indices, atypical atomic charge distributions (*AXp-2d*, *AETA_eta_F*), and divergent topological indices (*SpMax_A*, *Xc-5dv*) reflecting structural and electronic profiles that differ markedly from the main dataset. Overall, these findings indicate that molecular properties such as size, shape, electronic distribution, aromaticity, and the spatial arrangement of electronegative or polar groups play critical roles in nephrotoxicity. These properties can be mechanistically linked to kidney-specific toxic outcomes. For instance, polarity and dipole moment can influence renal tubular uptake and accumulation, while aromaticity may facilitate bioactivation to reactive metabolites associated with mitochondrial dysfunction or oxidative stress in kidney cells. These insights provide actionable guidance for optimizing molecular structures to reduce predicted nephrotoxic risk. In parallel, qRASAR models were developed using similarity and error-based descriptors. Among all models, the reduced EUC (Selected) model, using only three interpretable, error-based descriptors, offered a strong balance of accuracy (F1-score: 0.7327) and interpretability, emphasizing the utility of transparent models in regulatory contexts. The dataset size may represent a limitation of the present study. Future investigations could strengthen external validation by incorporating larger datasets (e.g., Tox21, PubChem bioassays data).

Finally, these efforts culminated in the deployment of “KidneyTox_v1.0”, an open-access, SHAP-integrated web platform for nephrotoxicity prediction. This tool supports hypothesis generation, compound prioritization (e.g., reducing overly electron-rich aromatic systems, modulating polarity, adjusting flexibility), and scaffold optimization in early drug discovery by offering visual, interpretable predictions for any query molecule. Importantly, “KidneyTox_v1.0” is designed for usability and sustainability. This tool will be updated periodically as new nephrotoxicity data become available, and users can upload proprietary compounds in standard SMILES formats. Uploaded data are processed in memory without being stored, ensuring user privacy and data anonymization.

## Methods

### Dataset

Drug-induced nephrotoxicity (*N*_*Total*_ = 565) dataset was sourced from literature^17,18^. Table S1 provides an overview of the selected nephrotoxic drugs and their associated toxicity profiles. The dataset comprises 565 chemically diverse small molecules, including 287 drugs reported to induce nephrotoxicity in humans (‘Toxic’) and 278 drugs with no known nephrotoxic effects (‘Non-toxic’).

### Chemical space analysis

Six common physicochemical descriptors namely octanol-water partition coefficient (*LogP*), molecular weight (*MW*), hydrogen bond acceptors (*HBA*), hydrogen bond donors (*HBD*), total number of ring systems in the molecule (*nRings*), number of rotatable bonds (*nRB*) were calculated by using the “RDKit” (https://www.rdkit.org/) cheminformatics toolkit (version rdkit-pypi-2022.9.5)^31^. Then bins plots were drawn to understand the chemical space of the investigated molecules.

After that “Fasda_v1.0” tool (https://github.com/Amincheminfom/Fasda_v1) was used to perform fingerprint-assisted scaffold diversity analysis of these investigated compounds associated with drug-induced nephrotoxicity. This fingerprint-assisted scaffold diversity analysis approach combines molecular fingerprinting, similarity analysis, dimensionality reduction, and clustering to understand the structural diversity and relationships within a dataset of molecules. Moreover, dividing the molecules into clusters helps in categorizing structurally similar compounds, which may be useful for tasks like scaffold analysis^19^.

### Machine learning model development

Mordred descriptors^20^ were calculated by using *in house* “Fiore_v1.0” platform (https://github.com/Amincheminfom/Fiore_v1.0). Features containing missing values, non-numeric entries, or quasi-constant behavior (defined as a single value appearing in more than 98% of samples) were excluded from further analysis. The objective was to clean the dataset and reduce the feature space by retaining only the most informative descriptors, thereby enhancing model performance, interpretability, and computational efficiency.

RFC were employed, with hyperparameter optimization conducted using Optuna (https://optuna.org/). The following RFC hyperparameters were tuned within the specified ranges: (i) number of estimators (*n_estimators*): 10–100; (ii) maximum tree depth (*max_depth*): 2–32; (iii) minimum number of samples required to split an internal node (*min_samples_split*): 2–16; and (iv) minimum number of samples required at a leaf node (*min_samples_leaf*): 1–16. The resulting RFC model were evaluated using statistical performance metrics as described earlier^21^.

### Read-across feature calculation

Once the AI-ML QSAR models were developed using molecular descriptors and fingerprints, the same feature spaces were used to generate similarity- and error-based RASAR descriptors. Optimal Read-Across hyperparameters were first identified by splitting the training set into sub-training and validation sets and generating validation predictions using Read-Across-v4.2.2 tool^32^. The number of close congeners (2–10) was systematically varied, and the optimal setting was selected based on validation performance. These optimized parameters were then used to compute RASAR descriptors for both training and test sets using RASAR-Desc-Calc-v3.0.3 tool^32^. The complete list of computed RASAR descriptors is provided in Table S2 of the Supplementary Material.

Like the QSAR analysis, feature selection was performed on the RASAR descriptor matrix to identify the most discriminating features. However, before this, we have deliberately removed the RASAR descriptors SD_Activity, SE, and CVact, which stands for the weighted standard deviation of the activity values of the close congeners, the corresponding standard error, and the coefficient of variation^29^. We employed the feature selection algorithm, i.e., identifying the most discriminating features, which aimed towards an unbiased feature selection due to its modeling algorithm-independent nature.

### qRASAR model development and validation

qRASAR classification models were constructed using similarity- and error-based read-across descriptors computed under three kernels: EUC, GK, and LK. For each kernel, two model families were developed: (i) Hybrid models combining the ML-selected molecular descriptors with kernel-specific RASAR descriptors, and (ii) RASAR-only models using only the error/similarity descriptors. This yielded six classification models in total (EUC-Hybrid, EUC-RASAR, GK-Hybrid, GK-RASAR, LK-Hybrid, LK-RASAR). External performance was assessed on the held-out test set using Accuracy, Precision, Recall, and F1. To enhance interpretability, feature importance ranking was applied and reduced (“Selected”) models were derived by retaining the top three error-based descriptors. Across kernels, three descriptors were consistently prioritized: MaxWtSim (maximum weighted similarity to analogs), MaxNeg (maximum similarity to known inactives), and gm (Banerjee-Roy coefficient), which captures neighborhood inconsistency via an error-weighted geometric mean. These error-aware, similarity-weighted descriptors distill the structural signal captured by the ML stage into chemically interpretable quantities. Among all six models, the EUC-Selected model showed the best overall performance, striking a strong balance between sensitivity and specificity while maintaining interpretability.

## Supplementary Information

Below is the link to the electronic supplementary material.

Supplementary Material 1

## Data Availability

Data are available in the manuscript, Supplementary material, and on the GitHub page (https://github.com/Amincheminform/KidneyTox_v1.0).
