# Supplementary material for: KidneyTox_v1.0 enables explainable artificial intelligence prediction of nephrotoxicity in small molecules
Source: Sci Rep. 2026 Jan 13;16:5099. doi: 10.1038/s41598-026-35496-4 (PMC12877123; doi:10.1038/s41598-026-35496-4)
Supplement: Supplementary file 1 — Supplementary Material 1 [file 41598_2026_35496_MOESM1_ESM.docx]

**KidneyTox_v1.0 Enables Explainable Artificial Intelligence Prediction of Nephrotoxicity in Small Molecules**

Sk. Abdul Amin^1*^, Supratik Kar^2*^, Stefano Piotto^1^

^1^Department of Pharmacy, University of Salerno,

Via Giovanni Paolo II 132, 84084 Fisciano, SA, Italy

^2^Chemometrics and Molecular Modeling Laboratory,

Department of Chemistry & Physics, Kean University,

1000 Morris Avenue, Union, NJ, 07083, USA

***Corresponding authors:** [pharmacist.amin@gmail.com](mailto:pharmacist.amin@gmail.com) (S.A. Amin), [skar@kean.edu](mailto:skar@kean.edu) (S. Kar).

**Table of Contents**

**Tables:**

**Table S1.** The list of selected nephrotoxic drugs and their associated toxicity profiles (1: ‘Toxic’, 0: ‘Non-toxic’).

**Table S2.** A comparison of the original RFC model with the top-performing models obtained from ten different random stratified splits.

**Table S3.** Detailed description of RASAR descriptors.

**Table S4.** Summary of the classification-based qRASAR models.

**Table S5**. Values of the descriptors of the outlier compounds.

**Figures:**

**Figure S1.** Elbow plot highlighted the relationship between the number of clusters (k) and the within-cluster sum of squares (WCSS). Here, the curve exhibits a noticeable “elbow” at k = 5, indicating that additional clusters beyond this point provide diminishing improvements in clustering performance. Therefore, five clusters were selected.

**Figure S2.** Accuracy across 50 independent stratified train-test splits (80:20). The highest-accuracy split (encircled black) was selected for downstream feature selection and model development.

**Figure S3.** (A) The plot of selected descriptors with their importance score. (B) The plot of applicability domain (AD) based on the leverage approach. The outliers (those outside AD) identified by leverage highlighted in blue circles.

**Table S1.** The list of selected nephrotoxic drugs and their associated toxicity profiles (1: ‘Toxic’, 0: ‘Non-toxic’).

| **Compound**  **ID** | **Smiles** | **Toxicity** | **Set** |
| --- | --- | --- | --- |
| 1 | CC1=C(CCN2CCC(CC2)C2=NOC3=CC(F)=CC=C23)C(=O)N2CCCCC2=N1 | 1 | Train |
| 2 | COC1=CC=NC(CS(=O)C2=NC3=CC=C(OC(F)F)C=C3N2)=C1OC | 1 | Train |
| 3 | CCCCN1C[C@H](O)[C@@H](O)[C@H](O)[C@H]1CO | 0 | Train |
| 4 | CCC(=C)C(=O)C1=CC=C(OCC(O)=O)C(Cl)=C1Cl | 0 | Train |
| 5 | CC1=C(N(N=C1C(=O)NN1CCCCC1)C1=CC=C(Cl)C=C1Cl)C1=CC=C(Cl)C=C1 | 0 | Train |
| 6 | CCCCC(=O)N(CC1=CC=C(C=C1)C1=C(C=CC=C1)C1=NNN=N1)[C@@H](C(C)C)C(O)=O | 1 | Train |
| 7 | C[C@H](CS)C(=O)N1CCC[C@H]1C(O)=O | 1 | Train |
| 8 | CC(C)N1CCN(CC1)C1=CC=C(OCC2COC(CN3C=NC=N3)(O2)C2=CC=C(Cl)C=C2Cl)C=C1 | 0 | Train |
| 9 | C\C(\C=C\C1=C(C)CCCC1(C)C)=C/C=C/C(/C)=C\C(O)=O | 1 | Train |
| 10 | OC[C@@H]1[C@@H](O)C[C@H](N2C=NC3=C2N=CNC3=O)C1=C | 1 | Train |
| 11 | CCN(CC)CCOC(=O)C1=CC=C(N)C=C1 | 0 | Test |
| 12 | OC1=C(Cl)C=C(Cl)C(Cl)=C1CC1=C(Cl)C(Cl)=CC(Cl)=C1O | 0 | Test |
| 13 | O[C@@H](CC[C@@H]1[C@H](N(C1=O)C1=CC=C(F)C=C1)C1=CC=C(O)C=C1)C1=CC=C(F)C=C1 | 1 | Train |
| 14 | OCC1OC(O)(CO)C(O)C1OC1OC(CO)C(O)C(O)C1O | 0 | Test |
| 15 | CCCCCCCN(CC)CCCC(O)C1=CC=C(NS(C)(=O)=O)C=C1 | 1 | Train |
| 16 | CNC(=O)CN(CCN(CCN(CC([O-])=O)CC(=O)NC)CC([O-])=O)CC([O-])=O | 1 | Train |
| 17 | C[N+](C)(C)CC([O-])=O | 0 | Train |
| 18 | FCOC(C(F)(F)F)C(F)(F)F | 1 | Train |
| 19 | COC1C(CC2CN3CCC4=C(NC5=CC(OC)=CC=C45)C3CC2C1C(=O)OC)OC(=O)C1=CC(OC)=C(OC)C(OC)=C1 | 0 | Train |
| 20 | COCC1=C(N2[C@H](SC1)[C@H](NC(=O)C(=N/OC)\C1=CSC(N)=N1)C2=O)C(O)=O | 1 | Test |
| 21 | C[C@@H]1C[C@H]2C3CCC4=CC(=O)C=C[C@]4(C)[C@@]3(Cl)[C@@H](O)C[C@]2(C)[C@@]1(O)C(=O)CCl | 0 | Test |
| 22 | COC1=CC=C2N=C(NC2=C1)S(=O)CC1=C(C)C(OC)=C(C)C=N1 | 1 | Train |
| 23 | CCCCCC(O)C=CC1C(O)CC2OC(CC12)=CCCCC(O)=O | 0 | Train |
| 24 | CN(N=O)C(=O)N[C@H]1[C@@H](O)O[C@H](CO)[C@@H](O)[C@@H]1O | 1 | Train |
| 25 | OCCOCCN1CCN(CC1)C1=NC2=C(SC3=C1C=CC=C3)C=CC=C2 | 1 | Test |
| 26 | COC1=CC2=C(C=C1OC)C(=O)C(CC1CCN(CC3=CC=CC=C3)CC1)C2 | 1 | Test |
| 27 | CC[C@H](C)C(=O)O[C@H]1C[C@@H](C)C=C2C=C[C@H](C)[C@H](CC[C@@H]3C[C@@H](O)CC(=O)O3)[C@@H]12 | 1 | Train |
| 28 | CC(=O)OC1=C(C=CC=C1)C(O)=O | 0 | Train |
| 29 | N#CC1=CC=C(C=C1)C(N1C=NC=N1)C1=CC=C(C=C1)C#N | 1 | Train |
| 30 | CC(C)OC(=O)OCOP(=O)(CO[C@H](C)CN1C=NC2=C(N)N=CN=C12)OCOC(=O)OC(C)C | 1 | Train |
| 31 | CN1C(=O)C(C)=C2N(C(=O)N(C3CC3)C(=O)C2=C1NC1=CC=C(I)C=C1F)C1=CC(NC(C)=O)=CC=C1 | 1 | Train |
| 32 | CN1C=CNC1=S | 0 | Train |
| 33 | OC(C(=O)OC1CC2CCC(C1)[N+]21CCCC1)(C1=CC=CC=C1)C1=CC=CC=C1 | 1 | Train |
| 34 | CCN(CC)CCNC(=O)C1=C(C)NC(\C=C2/C(=O)NC3=CC=C(F)C=C23)=C1C | 1 | Test |
| 35 | CCC[C@H](N[C@@H](C)C(=O)N1[C@H]2CCCC[C@H]2C[C@H]1C(O)=O)C(=O)OCC | 1 | Test |
| 36 | CCC(C)C1NC(=O)C(CC2=CC=C(O)C=C2)NC(=O)C(N)CSSCC(NC(=O)C(CC(N)=O)NC(=O)C(CCC(N)=O)NC1=O)C(=O)N1CCCC1C(=O)NC(CC(C)C)C(=O)NCC(N)=O | 0 | Train |
| 37 | CC(=O)OC1=CC2=C(CCN(C2)C(C(=O)C2CC2)C2=C(F)C=CC=C2)S1 | 0 | Train |
| 38 | CCC[C@H](N[C@@H](C)C(=O)N1[C@H]2CCCC[C@H]2C[C@H]1C(O)=O)C(O)=O | 1 | Test |
| 39 | CCCCCCC(C)(C)C1=CC2=C(C3CC(=O)CCC3C(C)(C)O2)C(O)=C1 | 0 | Test |
| 40 | CCCCCC(O)C=CC1C(O)CC(=O)C1CC=CCCCC(O)=O | 0 | Train |
| 41 | CN1CCN(CC1)C(=O)O[C@@H]1N(C(=O)C2=C1N=CC=N2)C1=CC=C(Cl)C=N1 | 1 | Train |
| 42 | C[NH+](C\C=C\C#CC(C)(C)C)CC1=C2C=CC=CC2=CC=C1 | 1 | Train |
| 43 | CN1CCN2C(C1)C1=C(CC3=C2N=CC=C3)C=CC=C1 | 1 | Train |
| 44 | CN1C=NC2=C1C(=O)N(C)C(=O)N2C | 1 | Train |
| 45 | ClC1=C(Cl)C2=C(C=C1)N=C1NC(=O)CN1C2 | 1 | Train |
| 46 | COCCCC\C(=N/OCC[NH3+])C1=CC=C(C=C1)C(F)(F)F | 1 | Train |
| 47 | OC1=CC=C(OCC2=CC=CC=C2)C=C1 | 0 | Train |
| 48 | CC1=C(O)C(C)=C2CCC(C)(COC3=CC=C(CC4SC(=O)NC4=O)C=C3)OC2=C1C | 0 | Train |
| 49 | CC(C)(C)NCC(O)COC1=C(C=CC=C1)C1CCCC1 | 0 | Test |
| 50 | COC1=C(N2CCNC(C)C2)C(F)=CC2=C1N(C=C(C(O)=O)C2=O)C1CC1 | 1 | Train |
| 51 | CC(C)NCC(O)COC1=C2C=CNC2=CC=C1 | 0 | Train |
| 52 | CCCC(=O)OC1(CCC2C3CCC4=CC(=O)CCC4(C)C3C(O)CC12C)C(=O)CO | 0 | Train |
| 53 | CCOC(=O)C1=C(COCCN)NC(C)=C(C1C1=C(Cl)C=CC=C1)C(=O)OC | 0 | Train |
| 54 | CCCCCCCCCCNCCN[C@@]1(C)C[C@H](O[C@@H]2[C@@H](O)[C@H](O)[C@@H](CO)O[C@H]2OC2=C3OC4=CC=C(C=C4Cl)[C@H](O)[C@@H](NC(=O)[C@@H](CC(C)C)NC)C(=O)N[C@@H](CC(N)=O)C(=O)N[C@@H]4C(C=C2OC2=C(Cl)C=C(C=C2)[C@@H](O)[C@@H]2NC(=O)[C@H](NC4=O)C4=CC=C(O)C(=C4)C4=C(C=C(O)C(CNCP(O)(O)=O)=C4O)[C@H](NC2=O)C(O)=O)=C3)O[C@@H](C)[C@H]1O | 1 | Train |
| 55 | C[C@H]1O[C@H](O[C@@H]2[C@@H](CO)O[C@H](O[C@@H]3[C@@H](CO)OC(O)[C@H](O)[C@H]3O)[C@H](O)[C@H]2O)[C@H](O)[C@@H](O)[C@@H]1N[C@H]1C=C(CO)[C@@H](O)[C@H](O)[C@H]1O | 0 | Train |
| 56 | CC1=CC=C(C=C1)S(=O)(=O)NC(=O)NN1CCCCCC1 | 0 | Test |
| 57 | OC1=CC2=C(C=C1)[C@H]([C@H](CC2)C1=CC=CC=C1)C1=CC=C(OCCN2CCCC2)C=C1 | 1 | Train |
| 58 | CCCCC1=NC(Cl)=C(CO)N1CC1=CC=C(C=C1)C1=C(C=CC=C1)C1=NNN=N1 | 1 | Train |
| 59 | COC1=CC(C)=C(C=CC(C)=CC=CC(C)=CC(O)=O)C(C)=C1C | 0 | Train |
| 60 | OC(=O)C1=C(OC(=O)C2=C(O)C=CC=C2)C=CC=C1 | 0 | Train |
| 61 | CC(C)C1=NC(CN(C)C(=O)N[C@@H](CCN2CCOCC2)C(=O)N[C@H](CC[C@H](CC2=CC=CC=C2)NC(=O)OCC2=CN=CS2)CC2=CC=CC=C2)=CS1 | 1 | Test |
| 62 | CCCCCOC1=CC=C(C=C1)C1=CC(=NO1)C1=CC=C(C=C1)C(=O)N[C@H]1C[C@@H](O)[C@@H](O)NC(=O)[C@@H]2[C@@H](O)[C@@H](C)CN2C(=O)[C@@H](NC(=O)[C@@H](NC(=O)[C@@H]2C[C@@H](O)CN2C(=O)[C@@H](NC1=O)[C@@H](C)O)[C@H](O)[C@@H](O)C1=CC=C(O)C(OS(O)(=O)=O)=C1)[C@H](O)CC(N)=O | 1 | Train |
| 63 | CC1CC2=C(C=CC=C2)N1NC(=O)C1=CC=C(Cl)C(=C1)S(N)(=O)=O | 1 | Test |
| 64 | COCC(=O)NC1=C(I)C(C(=O)N(C)CC(O)CO)=C(I)C(C(=O)NCC(O)CO)=C1I | 1 | Train |
| 65 | NCC1OC(OC2C(CO)OC(OC3C(O)C(N)CC(N)C3OC3OC(CN)C(O)C(O)C3N)C2O)C(N)C(O)C1O | 0 | Train |
| 66 | CCOC1=NC2=CC=CC(C(=O)OC(C)OC(=O)OC3CCCCC3)=C2N1CC1=CC=C(C=C1)C1=C(C=CC=C1)C1=NNN=N1 | 1 | Train |
| 67 | CC1=CC(=CC(C)=C1OC1=C(Br)C(N)=NC(NC2=CC=C(C=C2)C#N)=N1)C#N | 1 | Train |
| 68 | CC12CCC3C(CCC4=CC5=C(CC34C)C=NO5)C1CCC2(O)C#C | 0 | Train |
| 69 | CC(NC(C)(C)C)C(=O)C1=CC=CC(Cl)=C1 | 1 | Train |
| 70 | CC1=C(C=C(C#N)C(=O)N1)C1=CC=NC=C1 | 0 | Test |
| 71 | CCC(C)N1N=CN(C1=O)C1=CC=C(C=C1)N1CCN(CC1)C1=CC=C(OC[C@H]2CO[C@@](CN3C=NC=N3)(O2)C2=CC=C(Cl)C=C2Cl)C=C1 | 1 | Train |
| 72 | C[C@H]1OC(O[C@@H]2C[C@@H]3O[C@@](O)(C[C@H](O)[C@H]3C(O)=O)C[C@@H](O)C[C@H]3O[C@@H]3C=CC(=O)O[C@H](C)CC=CC=CC=CC=C2)[C@@H](O)[C@@H](N)[C@@H]1O | 0 | Train |
| 73 | CCCCCCCCCC(=O)N[C@@H](CC1=CNC2=CC=CC=C12)C(=O)N[C@@H](CC(N)=O)C(=O)N[C@@H](CC(O)=O)C(=O)NC1C(C)OC(=O)[C@H](CC(=O)C2=C(N)C=CC=C2)NC(=O)[C@@H](NC(=O)[C@@H](CO)NC(=O)CNC(=O)[C@H](CC(O)=O)NC(=O)[C@@H](C)NC(=O)[C@H](CC(O)=O)NC(=O)[C@H](CCCN)NC(=O)CNC1=O)C(C)CC(O)=O | 1 | Test |
| 74 | CCOC1=NC2=CC=CC(C(O)=O)=C2N1CC1=CC=C(C=C1)C1=C(C=CC=C1)C1=NNN=N1 | 1 | Train |
| 75 | ClC1=CC=C2OC(=O)NC2=C1 | 0 | Test |
| 76 | FC(F)OC(F)C(F)(F)F | 1 | Train |
| 77 | CCCCC(F)(F)[C@@]1(O)CC[C@H]2[C@@H](CC(=O)[C@@H]2CCCCCCC(O)=O)O1 | 0 | Train |
| 78 | CC1NC2=C(C=C(C(Cl)=C2)S(N)(=O)=O)C(=O)N1C1=C(C)C=CC=C1 | 0 | Train |
| 79 | CCCC1CC(N(C)C1)C(=O)NC(C(C)Cl)C1OC(SC)C(O)C(O)C1O | 0 | Train |
| 80 | CNC1(C)C2CCC(C2)C1(C)C | 0 | Train |
| 81 | CON=C(C(=O)N[C@H]1[C@H]2SCC(C=CC3=C(C)N=CS3)=C(N2C1=O)C(=O)OCOC(=O)C(C)(C)C)C1=CSC(N)=N1 | 1 | Train |
| 82 | NC1=CC=C(C=C1)S(=O)(=[OH+])[NH2+]C1=NC=CC=N1 | 0 | Train |
| 83 | CO[C@@H]1CC(CC[C@H]1O)\C=C(/C)[C@H]1OC(=O)[C@@H]2CCCCN2C(=O)C(=O)[C@]2(O)O[C@@H]([C@H](C[C@H]2C)OC)[C@H](C[C@@H](C)C\C(C)=C/[C@@H](CC=C)C(=O)C[C@H](O)[C@H]1C)OC | 1 | Train |
| 84 | CC(C)(C)NC(=O)C1CCC2C3CCC4NC(=O)C=CC4(C)C3CCC12C | 0 | Train |
| 85 | NC1=C2N=CN(CCOCP(O)(O)=O)C2=NC=N1 | 1 | Train |
| 86 | COC1=C(C)C2=C(C(=O)OC2)C(O)=C1C\C=C(/C)CCC(O)=O | 1 | Train |
| 87 | NNC1=NN=CC2=CC=CC=C12 | 1 | Train |
| 88 | COCCCN1CCC(CC1)NC(=O)C1=C2OCCC2=C(N)C(Cl)=C1 | 0 | Train |
| 89 | CC(N)C(O)C1=CC=CC=C1 | 0 | Train |
| 90 | CCC(C)C1NC(=O)C(CC2=CC=C(O)C=C2)NC(=O)C(N)CSSCC(NC(=O)C(CC(N)=O)NC(=O)C(NC1=O)C(C)O)C(=O)N1CCCC1C(=O)NC(CC(C)C)C(=O)NCC(N)=O | 0 | Test |
| 91 | CN(C)CCC[C@]1(OCC2=C1C=CC(=C2)C#N)C1=CC=C(F)C=C1 | 1 | Train |
| 92 | CC(C)C1=C(\C=C\[C@@H](O)C[C@@H](O)CC(O)=O)C(=NC(=N1)N(C)S(C)(=O)=O)C1=CC=C(F)C=C1 | 1 | Test |
| 93 | CCC(C)C(=O)OC1CC(C)C=C2C=CC(C)C(CCC3CC(O)CC(=O)O3)C12 | 0 | Train |
| 94 | CC(=O)NC1C(O)OC(COS(O)(=O)=O)C(OC2OC(C(OC3OC(CO)C(OC4OC(C(O)C(O)C4OS(O)(=O)=O)C(O)=O)C(OS(O)(=O)=O)C3NS(O)(=O)=O)C(O)C2OS(O)(=O)=O)C(O)=O)C1O | 0 | Train |
| 95 | CCC1=C(C)CN(C(=O)NCCC2=CC=C(C=C2)S(=O)(=O)NC(=O)NC2CCC(C)CC2)C1=O | 0 | Test |
| 96 | CCCNC(C)C(=O)NC1=C(SC=C1C)C(=O)OC | 0 | Train |
| 97 | C[C@@H]1CCN([C@H](C1)C(O)=O)C(=O)[C@H](CCCN=C(N)N)NS(=O)(=O)C1=C2NCC(C)CC2=CC=C1 | 1 | Train |
| 98 | CN1C(C(=O)NC2=NC=C(C)S2)=C([O-])C2=C(C=CC=C2)S1(=O)=O | 1 | Train |
| 99 | CCCCCCCCCCNCC=C | 0 | Train |
| 100 | NC1CSSCC(NC(=O)C(CC(N)=O)NC(=O)C(CCC(N)=O)NC(=O)C(CC2=CC=CC=C2)NC(=O)C(CC2=CC=C(O)C=C2)NC1=O)C(=O)N1CCCC1C(=O)NC(CCCN=C(N)N)C(=O)NCC(N)=O | 0 | Train |
| 101 | CCC[C@H](NC(=O)[C@@H]1[C@H]2CCC[C@H]2CN1C(=O)[C@@H](NC(=O)[C@@H](NC(=O)C1=CN=CC=N1)C1CCCCC1)C(C)(C)C)C(=O)C(=O)NC1CC1 | 1 | Train |
| 102 | NC1=NC(=O)N(C=C1)[C@@H]1CS[C@H](CO)O1 | 1 | Test |
| 103 | CC1=CC=C(C=C1)N(CC1=NCCN1)C1=CC=CC(O)=C1 | 0 | Train |
| 104 | NC12CC3CC(CC(C3)C1)C2 | 1 | Test |
| 105 | COC1=CC2=NC(=NC(N)=C2C=C1OC)N(C)CCCNC(=O)C1CCCO1 | 1 | Train |
| 106 | CN1C=NC2=C1C(=O)N(CCCCC(C)=O)C(=O)N2C | 0 | Train |
| 107 | CCCSC1=CC=C2N=C(NC(=O)OC)NC2=C1 | 1 | Train |
| 108 | CCCC1OC2CC3C4CCC5=CC(=O)C=CC5(C)C4C(O)CC3(C)C2(O1)C(=O)CO | 0 | Test |
| 109 | FC(F)OC(F)(F)C(F)Cl | 0 | Train |
| 110 | CC(C)(N)CC1=CC=CC=C1 | 0 | Test |
| 111 | NC1=NC2=NC=C(CC(CC#C)C3=CC=C(C=C3)C(=O)N[C@@H](CCC(O)=O)C(O)=O)N=C2C(N)=N1 | 1 | Train |
| 112 | CC1(C)OC2CC3C4CCC5=CC(=O)CCC5(C)C4(F)C(O)CC3(C)C2(O1)C(=O)CCl | 0 | Test |
| 113 | [O-][N+](=O)C1=CC(=CC=C1C(=O)C1C(=O)CCCC1=O)C(F)(F)F | 0 | Train |
| 114 | C[C@@H](NCCCC1=CC=CC(=C1)C(F)(F)F)C1=C2C=CC=CC2=CC=C1 | 1 | Train |
| 115 | COC1=CC=C2CC3C4CCC(O)C5OC1=C2C45CCN3C | 0 | Test |
| 116 | CC1(C)OC2CC3C4CC(F)C5=CC(=O)CCC5(C)C4C(O)CC3(C)C2(O1)C(=O)CO | 0 | Train |
| 117 | CCCC(=O)NC1=CC=C(OCC(O)CNC(C)C)C(=C1)C(C)=O | 0 | Train |
| 118 | CC(=O)O[C@@]12CO[C@@H]1C[C@H](O)[C@]1(C)[C@@H]2[C@H](OC(=O)C2=CC=CC=C2)[C@]2(O)C[C@H](OC(=O)[C@H](O)[C@@H](NC(=O)OC(C)(C)C)C3=CC=CC=C3)C(C)=C([C@@H](O)C1=O)C2(C)C | 1 | Train |
| 119 | CC([O-])=O | 0 | Test |
| 120 | COC1=CC2=C(C(OC)=C1OC)C1=CC=C(OC)C(=O)C=C1[C@H](CC2)NC(C)=O | 1 | Test |
| 121 | OC1=C(C2CCC(CC2)C2=CC=C(Cl)C=C2)C(=O)C(=O)C2=C1C=CC=C2 | 1 | Train |
| 122 | CCC(C)CCCCC(=O)NC(CCN)C(=O)NC(C(C)O)C(=O)NC(CCN)C(=O)NC1CCNC(=O)C(NC(=O)C(CCN)NC(=O)C(CCN)NC(=O)C(CC(C)C)NC(=O)C(CC2=CC=CC=C2)NC(=O)C(CCN)NC1=O)C(C)O | 0 | Train |
| 123 | CCN(CC)C(=O)[C@@]1(C[C@@H]1C[NH3+])C1=CC=CC=C1 | 1 | Test |
| 124 | CO\N=C(/C(=O)N[C@H]1[C@H]2SCC(C[N+]3(C)CCCC3)=C(N2C1=O)C([O-])=O)C1=CSC(N)=N1 | 1 | Train |
| 125 | CC(C)(C)C1=NC(=C(S1)C1=CC=NC(N)=N1)C1=CC=CC(NS(=O)(=O)C2=C(F)C=CC=C2F)=C1F | 1 | Train |
| 126 | CCCCC1=C(C(=O)C2=CC(I)=C(OCC[NH+](CC)CC)C(I)=C2)C2=CC=CC=C2O1 | 1 | Train |
| 127 | COC1=C2O[C@H]3C[C@@H](O)C=C[C@]33CCN(C)CC(C=C1)=C23 | 1 | Train |
| 128 | NC[C@H]1O[C@H](O[C@@H]2[C@@H](N)C[C@@H](N)[C@H](O[C@H]3O[C@H](CO)[C@@H](O)[C@H](N)[C@H]3O)[C@H]2O)[C@H](N)C[C@@H]1O | 1 | Train |
| 129 | C[N+](C)(C)C[C@H](O)CC([O-])=O | 1 | Train |
| 130 | ClC1=CC=C(CCC(CN2C=CN=C2)SC2=C(Cl)C=CC=C2Cl)C=C1 | 0 | Train |
| 131 | CC(COC1=CC=CC=C1)NC(C)C(O)C1=CC=C(O)C=C1 | 0 | Train |
| 132 | CCOC(=O)C1=CC=C(N=C1)C#CC1=CC=C2SCCC(C)(C)C2=C1 | 0 | Train |
| 133 | NC(CC1=CC(I)=C(OC2=CC(I)=C(O)C(I)=C2)C(I)=C1)C(O)=O | 0 | Train |
| 134 | CCN(CC)CCNC(=O)C1=C(OC)C=C(N)C(Cl)=C1 | 0 | Train |
| 135 | ClC1=CC=C(C(CN2C=CN=C2)OCC2=CSC3=C(Cl)C=CC=C23)C(Cl)=C1 | 0 | Train |
| 136 | CC(C)[C@H](N)C(=O)OCC(CO)OCN1C=NC2=C1N=C(N)NC2=O | 1 | Test |
| 137 | CCN(C)C(=O)OC1=CC(=CC=C1)[C@H](C)N(C)C | 1 | Train |
| 138 | CN=C(NCCSCC1=C(C)NC=N1)NC#N | 1 | Test |
| 139 | OC[C@@H]1CC[C@@H](O1)N1C=NC2=C1N=CNC2=O | 1 | Train |
| 140 | COC1=CC=C(OC)C(=C1)C(O)CNC(=O)CN | 0 | Train |
| 141 | CCCCCOC1=CC=C(C=C1)C1=CC(=NO1)C1=CC=C(C=C1)C(=O)NC1C[C@H](O)[C@@H](O)NC(=O)[C@H]2[C@H](O)[C@H](C)CN2C(=O)[C@H](NC(=O)[C@H](NC(=O)[C@H]2C[C@H](O)CN2C(=O)[C@H](NC1=O)[C@@H](C)O)C(O)[C@@H](O)C1=CC=C(O)C(OS(O)(=O)=O)=C1)[C@@H](O)CC(N)=O | 1 | Train |
| 142 | CO\N=C(/C(=O)N[C@H]1[C@H]2SCC(CSC3=NC(=O)C(=O)NN3C)=C(N2C1=O)C(O)=O)C1=CSC(N)=N1 | 1 | Train |
| 143 | C[C@H](C1=CN=CN1)C1=C(C)C(C)=CC=C1 | 1 | Train |
| 144 | C[C@@H]1C[C@H]2C3C[C@H](F)C4=CC(=O)C=C[C@]4(C)[C@@]3(F)[C@@H](O)C[C@]2(C)[C@@]1(O)C(=O)SCF | 0 | Test |
| 145 | NCC1OC(OC2C(CO)OC(OC3C(O)C(N)CC(N)C3OC3OC(CO)C(O)C(O)C3N)C2O)C(N)C(O)C1O | 0 | Train |
| 146 | CC1=C(CCO)SC=[N+]1CC1=C(N)N=C(C)N=C1 | 0 | Test |
| 147 | CC1=[N+]([O-])C=C(N=C1)C(O)=O | 0 | Train |
| 148 | CN1N=C(C(=O)NC2CC3CCCC(C2)N3C)C2=CC=CC=C12 | 0 | Train |
| 149 | CC[C@]12CC(=C)[C@H]3[C@@H](CCC4=CCCC[C@H]34)[C@@H]1CC[C@@]2(O)C#C | 0 | Test |
| 150 | OC1=CC2=C(CC3N(CC4CCC4)CCC22CCCCC32O)C=C1 | 0 | Train |
| 151 | CNC(NCCSCC1=CC=C(CN(C)C)O1)=C[N+]([O-])=O | 0 | Train |
| 152 | CC1(C)O[C@@H]2CO[C@@]3(COS(N)(=O)=O)OC(C)(C)O[C@H]3[C@@H]2O1 | 1 | Train |
| 153 | CC(C)(CO)C(O)C(=O)NCCCO | 0 | Test |
| 154 | CC(C)(C)NC(=O)[C@@H]1CN(CC2=CC=CN=C2)CCN1C[C@@H](O)C[C@@H](CC1=CC=CC=C1)C(=O)N[C@@H]1[C@H](O)CC2=C1C=CC=C2 | 1 | Train |
| 155 | OC(CC=O)C=O | 0 | Test |
| 156 | COC(=O)N[C@H](C(=O)N[C@@H](CC1=CC=CC=C1)[C@@H](O)CN(CC1=CC=C(C=C1)C1=NC=CC=C1)NC(=O)[C@@H](NC(=O)OC)C(C)(C)C)C(C)(C)C | 1 | Train |
| 157 | CCCCCOC(=O)NC1=NC(=O)N(C=C1F)[C@@H]1O[C@H](C)[C@@H](O)[C@H]1O | 1 | Train |
| 158 | OC1CCC2(O)C3CC4=CC=C(O)C5=C4C2(CCN3CC2CCC2)C1O5 | 0 | Train |
| 159 | CNS(=O)(=O)CCC1=CC=C2NC=C(C3CCN(C)CC3)C2=C1 | 1 | Train |
| 160 | CC1=C(C(=O)N[C@H]2[C@H]3SC(C)(C)[C@@H](N3C2=O)C(O)=O)C(=NO1)C1=C(Cl)C=CC=C1Cl | 1 | Train |
| 161 | CCN1CCN(C(=O)N[C@@H](C(=O)N[C@H]2[C@H]3SC(C)(C)[C@@H](N3C2=O)C(O)=O)C2=CC=CC=C2)C(=O)C1=O | 1 | Test |
| 162 | CC(C)[C@H](N)C(=O)OCCOCN1C=NC2=C1N=C(N)NC2=O | 1 | Train |
| 163 | COC(=O)[C@@H](N1CCC2=C(C1)C=CS2)C1=C(Cl)C=CC=C1 | 1 | Train |
| 164 | CCCC1=NC(=C(N1CC1=CC=C(C=C1)C1=C(C=CC=C1)C1=NNN=N1)C(O)=O)C(C)(C)O | 1 | Train |
| 165 | COC1=C(OC)C=C2C(N)=NC(=NC2=C1)N1CCN(CC1)C(=O)C1CCCO1 | 0 | Train |
| 166 | COC1=C2N=CN([C@@H]3O[C@H](CO)[C@@H](O)[C@@H]3O)C2=NC(N)=N1 | 1 | Train |
| 167 | C[N+]1(C)CCCC(C1)OC(=O)C(O)(C1=CC=CC=C1)C1=CC=CC=C1 | 0 | Train |
| 168 | OCCN(CCO)C1=NC(N2CCCCC2)=C2N=C(N=C(N3CCCCC3)C2=N1)N(CCO)CCO | 1 | Train |
| 169 | CSCCC1NC(=O)C(CC(C)C)NC(=O)C(CCCNC(N)=N)NC(=O)C(CO)NC(=O)C2CSSCC3NC(=O)C(CO)NC(=O)CNC(=O)C(NC(=O)C(CSSCC(N)C(=O)NC(CCCCN)C(=O)NCC(=O)NC(CCCCN)C(=O)NCC(=O)NC(C)C(=O)NC(CCCCN)C(=O)N2)NC(=O)C(CSSCC(NC(=O)C(CCCCN)NC(=O)CNC(=O)C(CO)NC(=O)C(CCCNC(N)=N)NC3=O)C(N)=O)NC(=O)C(CC(O)=O)NC(=O)C(CC2=CC=C(O)C=C2)NC1=O)C(C)O | 1 | Test |
| 170 | CN1CCN(CC2=CC=C(C=C2)C(=O)NC2=CC=C(C)C(NC3=NC(=CC=N3)C3=CC=CN=C3)=C2)CC1 | 1 | Test |
| 171 | NCCS | 1 | Test |
| 172 | CS(C)=O | 0 | Train |
| 173 | CC1CC2C3CC(F)C4=CC(=O)C=CC4(C)C3(F)C(O)CC2(C)C1(O)C(=O)CO | 0 | Train |
| 174 | CCCCC1=NC2(CCCC2)C(=O)N1CC1=CC=C(C=C1)C1=C(C=CC=C1)C1=NNN=N1 | 1 | Test |
| 175 | CC1(C)SC2C(NC(=O)CC3=CC=CC=C3)C(=O)N2C1C(O)=O | 0 | Train |
| 176 | CC[C@H](C)[C@H](NC(=O)[C@H](CC(C)C)NC(=O)[C@H](CO)NC(=O)[C@H](CC1=CN=CN1)NC(=O)[C@@H](NC(=O)[C@H](CC(C)C)NC(=O)[C@H](CO)NC(=O)[C@@H](NC(=O)[C@H](CC1=CC=C(O)C=C1)NC(C)=O)[C@@H](C)O)[C@@H](C)CC)C(=O)N[C@@H](CCC(O)=O)C(=O)N[C@@H](CCC(O)=O)C(=O)N[C@@H](CO)C(=O)N[C@@H](CCC(N)=O)C(=O)N[C@@H](CC(N)=O)C(=O)N[C@@H](CCC(N)=O)C(=O)N[C@@H](CCC(N)=O)C(=O)N[C@@H](CCC(O)=O)C(=O)N[C@@H](CCCCN)C(=O)N[C@@H](CC(N)=O)C(=O)N[C@@H](CCC(O)=O)C(=O)N[C@@H](CCC(N)=O)C(=O)N[C@@H](CCC(O)=O)C(=O)N[C@@H](CC(C)C)C(=O)N[C@@H](CC(C)C)C(=O)N[C@@H](CCC(O)=O)C(=O)N[C@@H](CC(C)C)C(=O)N[C@@H](CC(O)=O)C(=O)N[C@@H](CCCCN)C(=O)N[C@@H](CC1=CNC2=CC=CC=C12)C(=O)N[C@@H](C)C(=O)N[C@@H](CO)C(=O)N[C@@H](CC(C)C)C(=O)N[C@@H](CC1=CNC2=CC=CC=C12)C(=O)N[C@@H](CC(N)=O)C(=O)N[C@@H](CC1=CNC2=CC=CC=C12)C(=O)N[C@@H](CC1=CC=CC=C1)C(N)=O | 1 | Train |
| 177 | C(OC1=CC=C2OCOC2=C1)[C@@H]1CNCC[C@H]1C1=CC=CC=C1 | 1 | Train |
| 178 | CCNC(=O)N(CCCN(C)C)C(=O)C1CC2C(CC3=CNC4=CC=CC2=C34)N(CC=C)C1 | 0 | Train |
| 179 | COC1=CC=C(C=C1C12CC3CC(CC(C3)C1)C2)C1=CC=C2C=C(C=CC2=C1)C(O)=O | 0 | Train |
| 180 | CC1=CC(\C=C\C#N)=CC(C)=C1NC1=CC=NC(NC2=CC=C(C=C2)C#N)=N1 | 1 | Test |
| 181 | NC1=NC2=CC=C(OC(F)(F)F)C=C2S1 | 1 | Train |
| 182 | CC(=O)N1CCN(CC1)C1=CC=C(OCC2COC(CN3C=CN=C3)(O2)C2=CC=C(Cl)C=C2Cl)C=C1 | 0 | Test |
| 183 | NCC1=CC=C(C=C1)S(N)(=O)=O | 0 | Train |
| 184 | CCCN(CCOC1=C(Cl)C=C(Cl)C=C1Cl)C(=O)N1C=CN=C1 | 1 | Train |
| 185 | CC(C)(C)NC(=O)[C@@H]1C[C@@H]2CCCC[C@@H]2CN1C[C@@H](O)[C@H](CC1=CC=CC=C1)NC(=O)[C@H](CC(N)=O)NC(=O)C1=CC=C2C=CC=CC2=N1 | 1 | Train |
| 186 | CCCCCC(O)C=CC1C(O)CC(O)C1CC=CCCCC(O)=O | 0 | Train |
| 187 | CCCNCC(O)COC1=C(C=CC=C1)C(=O)CCC1=CC=CC=C1 | 1 | Train |
| 188 | CC(C)CN(C[C@@H](OP([O-])([O-])=O)[C@H](CC1=CC=CC=C1)NC(=O)O[C@H]1CCOC1)S(=O)(=O)C1=CC=C(N)C=C1 | 1 | Train |
| 189 | OC1=CC=C(C=C1)C1=C(C(=O)C2=CC=C(OCCN3CCCCC3)C=C2)C2=CC=C(O)C=C2S1 | 0 | Train |
| 190 | CCOC(=O)[C@H](CCC1=CC=CC=C1)N[C@@H](C)C(=O)N1[C@H]2CCC[C@H]2C[C@H]1C(O)=O | 1 | Test |
| 191 | CC12CCC3C(CCC4=C3C=CC(O)=C4)C1CCC2O | 0 | Train |
| 192 | CC(C)[C@H](NC(=O)N(C)CC1=CSC(=N1)C(C)C)C(=O)N[C@H](C[C@H](O)[C@H](CC1=CC=CC=C1)NC(=O)OCC1=CN=CS1)CC1=CC=CC=C1 | 1 | Train |
| 193 | OC(=O)C1=CC(=CC=C1O)C1=CC=C(F)C=C1F | 1 | Train |
| 194 | CC1=C(CCC(O)=O)/C2=C/C3=C(CCC(O)=O)C(C)=C([N-]3)\C=C3/N=C(/C=C4\N=C(\C=C\1/[N-]\2)C(C)=C4C=C)C(C)=C3C=C | 1 | Test |
| 195 | CC[C@H]1OC(=O)[C@H](C)C(=O)[C@H](C)[C@@H](O[C@@H]2O[C@H](C)C[C@@H]([C@H]2O)N(C)C)[C@@](C)(C[C@@H](C)C(=O)[C@@H](C)[C@H]2N(CCCCN3C=NC(=C3)C3=CC=CN=C3)C(=O)O[C@]12C)OC | 0 | Test |
| 196 | NC1=CC=C(N=NC2=CC=CC=C2)C(N)=N1 | 0 | Train |
| 197 | NCCCC(O)=O | 0 | Train |
| 198 | CC1CC2C3CCC4=CC(=O)C=CC4(C)C3(F)C(O)CC2(C)C1(O)C(=O)CO | 0 | Test |
| 199 | C(NC1=C2NC=NC2=NC=N1)C1=CC=CO1 | 0 | Train |
| 200 | C1C2CNCC1C1=C2C=C2N=CC=NC2=C1 | 1 | Test |
| 201 | NC(=O)CCC1NC(=O)C(CC2=CC=CC=C2)NC(=O)C(CC2=CC=C(O)C=C2)NC(=O)CCSSCC(NC(=O)C(CC(N)=O)NC1=O)C(=O)N1CCCC1C(=O)NC(CCCN=C(N)N)C(=O)NCC(N)=O | 0 | Train |
| 202 | ClCCN(CCCl)P1(=O)NCCCO1 | 1 | Test |
| 203 | NC1=CC(=NC(=N)N1O)N1CCCCC1 | 0 | Train |
| 204 | COC1=CC(=CC(OC)=C1O)[C@H]1[C@@H]2[C@H](COC2=O)[C@H](O[C@@H]2O[C@@H]3CO[C@H](O[C@H]3[C@H](O)[C@H]2O)C2=CC=CS2)C2=C1C=C1OCOC1=C2 | 1 | Train |
| 205 | OC12CC3CC(C1)CC(C3)(C2)NCC(=O)N1CCCC1C#N | 0 | Test |
| 206 | COC1=CC=C2[N-]C(=NC2=C1)[S@@](=O)CC1=C(C)C(OC)=C(C)C=N1 | 1 | Train |
| 207 | COC1=CC2=CC=C(C=C2C=C1)[C@H](C)C(O)=O | 1 | Train |
| 208 | [O-]C(=O)P([O-])([O-])=O | 1 | Train |
| 209 | OC(=O)C1=CC=C(C=C1)N1N=C(N=C1C1=C(O)C=CC=C1)C1=C(O)C=CC=C1 | 1 | Train |
| 210 | CC1C2CC3=C(C=C(O)C=C3)C1(C)CCN2CC=C(C)C | 0 | Test |
| 211 | NC1=CC=C(C=C1)S(=O)(=O)NC1=NC=CC=N1 | 0 | Train |
| 212 | CCN1C=C(C(O)=O)C(=O)C2=C1C(F)=C(N1CCNC(C)C1)C(F)=C2 | 1 | Test |
| 213 | CC(C)NCC(O)C1=CC=C(O)C(O)=C1 | 0 | Train |
| 214 | CO[C@H]1O[C@H](COS([O-])(=O)=O)[C@@H](O[C@@H]2O[C@H]([C@@H](O[C@H]3O[C@H](COS([O-])(=O)=O)C(O[C@@H]4O[C@@H]([C@@H](O[C@H]5O[C@H](COS([O-])(=O)=O)[C@@H](O)[C@H](O)[C@H]5NS([O-])(=O)=O)[C@H](O)[C@H]4O)C([O-])=O)[C@H](OS([O-])(=O)=O)[C@H]3NS([O-])(=O)=O)[C@H](O)[C@H]2OS([O-])(=O)=O)C([O-])=O)[C@H](O)[C@H]1NS([O-])(=O)=O | 0 | Test |
| 215 | C[C@]12CCC3[C@@H](CC[C@H]4NC(=O)C=C[C@]34C)[C@@H]1CC[C@@H]2C(=O)NC1=CC(=CC=C1C(F)(F)F)C(F)(F)F | 0 | Train |
| 216 | NC1=NC(=CS1)C(=C\CC(O)=O)\C(=O)N[C@H]1[C@H]2SCC=C(N2C1=O)C(O)=O | 1 | Test |
| 217 | CC12CC(O)C3C(CCC4=CC(=O)CCC34C)C1CCC2(O)C(=O)CO | 0 | Train |
| 218 | CC(C)CCCC(C)CCCC(C)CCCC1(C)CCC2=C(C)C(OC(=O)CCC(=O)OCCO)=C(C)C(C)=C2O1 | 0 | Train |
| 219 | CCN(CC)C(=O)NC1=CC=C(OCC(O)CNC(C)(C)C)C(=C1)C(C)=O | 0 | Train |
| 220 | CCCCC(C)(C)C(O)C=CC1C(O)CC(=O)C1CCCCC=CC(=O)OC | 0 | Train |
| 221 | COC1=CC2=C(C=C1OC)[C@@H](CN(C)CCCN1CCC3=C(CC1=O)C=C(OC)C(OC)=C3)C2 | 0 | Train |
| 222 | CC(N)(CC1=CC=C(O)C(O)=C1)C(O)=O | 0 | Train |
| 223 | O=C1N(C2CCC(=O)NC2=O)C(=O)C2=C1C=CC=C2 | 1 | Train |
| 224 | CN1CCCC1CCOC(C)(C1=CC=CC=C1)C1=CC=C(Cl)C=C1 | 0 | Test |
| 225 | CC(C)CC(NC(=O)CNC(=O)C(CC1=CC=C(O)C=C1)NC(=O)C(CO)NC(=O)C(CC1=CNC2=CC=CC=C12)NC(=O)C(CC1=CN=CN1)NC(=O)C1CCC(=O)N1)C(=O)NC(CCCN=C(N)N)C(=O)N1CCCC1C(=O)NCC(N)=O | 0 | Train |
| 226 | NC(=O)C1CCCN1C(=O)C(CC1=CN=CN1)NC(=O)C1CCC(=O)N1 | 0 | Test |
| 227 | NC1=NC2=C(N=CN2COCCO)C(=O)N1 | 1 | Train |
| 228 | CC(=O)C1(O)CCC2C3C=C(Cl)C4=CC(=O)C5CC5C4(C)C3CCC12C | 0 | Train |
| 229 | CCCCCCCCCC(=O)NC(CC1=CNC2=CC=CC=C12)C(=O)NC(CC(N)=O)C(=O)NC(CC(O)=O)C(=O)NC1C(C)OC(=O)C(CC(=O)C2=C(N)C=CC=C2)NC(=O)C(NC(=O)C(CO)NC(=O)CNC(=O)C(CC(O)=O)NC(=O)C(C)NC(=O)C(CC(O)=O)NC(=O)C(CCCN)NC(=O)CNC1=O)C(C)CC(O)=O | 1 | Train |
| 230 | CO\N=C(/C(=O)N[C@H]1[C@H]2SCC(COC(N)=O)=C(N2C1=O)C(O)=O)C1=CC=CO1 | 1 | Train |
| 231 | CC(C)(O\N=C(/C(=O)N[C@H]1[C@H]2SCC(C[N+]3=CC=CC=C3)=C(N2C1=O)C([O-])=O)C1=CSC(N)=N1)C(O)=O | 1 | Test |
| 232 | CC(=O)OC[C@H]1O[C@@H]([S-])[C@H](OC(C)=O)[C@@H](OC(C)=O)[C@@H]1OC(C)=O | 1 | Train |
| 233 | OC(=O)C1=CC(=CC=C1O)N=NC1=CC=C(O)C(=C1)C(O)=O | 1 | Train |
| 234 | CCCC(C)(COC(N)=O)COC(=O)NC(C)C | 0 | Train |
| 235 | CCCCCCCCCCC[C@@H](C[C@@H]1OC(=O)[C@H]1CCCCCC)OC(=O)[C@H](CC(C)C)NC=O | 1 | Test |
| 236 | COC1=CC=C2N=C(NC2=C1)[S@](=O)CC1=C(C)C(OC)=C(C)C=N1 | 1 | Train |
| 237 | NC1=C2N=CN([C@H]3C[C@H](O)[C@@H](CO)O3)C2=NC(Cl)=N1 | 1 | Train |
| 238 | OC(=O)CC[Ge](=O)O[Ge](=O)CCC(O)=O | 1 | Train |
| 239 | C[C@@H](O)[C@@H]1[C@H]2[C@@H](C)C(S[C@@H]3CN[C@H](CNS(N)(=O)=O)C3)=C(N2C1=O)C(O)=O | 0 | Test |
| 240 | CC(C)CC1N2C(=O)C(NC(=O)C3CN(C)C4CC5=C(Br)NC6=CC=CC(=C56)C4=C3)(OC2(O)C2CCCN2C1=O)C(C)C | 0 | Test |
| 241 | N[C@@H](C(=O)N[C@H]1[C@H]2SCC(Cl)=C(N2C1=O)C(O)=O)C1=CC=CC=C1 | 1 | Train |
| 242 | N[C@H](CCCN=C(N)N)C(=O)NC(CCCN=C(N)N)C(=O)N1CCCC1C(=O)N1C[C@H](O)CC1C(=O)NCC(=O)N[C@@H](CC1=CC=CS1)C(=O)N[C@@H](CO)C(=O)N1CC2=C(CC1C(=O)N1[C@H]3CCCC[C@H]3CC1C(=O)N[C@@H](CCCN=C(N)N)C(O)=O)C=CC=C2 | 0 | Train |
| 243 | CC(N)C12CC3CC(CC(C3)C1)C2 | 0 | Train |
| 244 | CC1=C(C=C2C(=C1)C(C)(C)CCC2(C)C)C(=C)C1=CC=C(C=C1)C(O)=O | 1 | Train |
| 245 | CC1=CC(NC(=O)C2=C(C)C=CC=C2)=CC=C1C(=O)N1CCCC(O)C2=C1C=CC(Cl)=C2 | 1 | Train |
| 246 | CN1C2=C(C3=CC=CC=C13)C(=O)N(CC1=C(C)NC=N1)CC2 | 0 | Test |
| 247 | NCCCC(N)(C(F)F)C(O)=O | 0 | Train |
| 248 | C[C@H]1CN(C[C@@H](C)N1)C1=C(F)C2=C(C(N)=C1F)C(=O)C(=CN2C1CC1)C(O)=O | 1 | Train |
| 249 | CC1COC2=C(N3CCN(C)CC3)C(F)=CC3=C2N1C=C(C(O)=O)C3=O | 1 | Test |
| 250 | CN(C)C(=N)N=C(N)N | 0 | Train |
| 251 | CC(N)COC1=C(C)C=CC=C1C | 0 | Train |
| 252 | N[C@@H](CC(=O)N1CCN2C(C1)=NN=C2C(F)(F)F)CC1=C(F)C=C(F)C(F)=C1 | 1 | Train |
| 253 | CC1CC(=O)NN=C1C1=CC=C(NN=C(C#N)C#N)C=C1 | 0 | Train |
| 254 | CC1=C(O)C(CO)=C(CO)C=N1 | 0 | Test |
| 255 | COC(=O)C1=C(C)NC(C)=C(C1C1=CC=CC2=NON=C12)C(=O)OC(C)C | 0 | Train |
| 256 | CC(O)C(C)C1OC1CC1COC(CC(C)=CC(=O)OCCCCCCCCC(O)=O)C(O)C1O | 0 | Test |
| 257 | CO\N=C1/CN(CC1CN)C1=NC2=C(C=C1F)C(=O)C(=CN2C1CC1)C(O)=O | 1 | Train |
| 258 | C[C@]1(O)CC[C@H]2[C@@H]3CC[C@H]4CC(=O)OC[C@]4(C)[C@H]3CC[C@]12C | 0 | Train |
| 259 | O[C@H](CC=O)C=O | 1 | Test |
| 260 | NC(CCCN=C(N)N)C(O)=O | 0 | Train |
| 261 | CCN1C=C(C(O)=O)C(=O)C2=C1C=C(N1CCNCC1)C(F)=C2 | 1 | Train |
| 262 | CCC(=O)OCC(=O)C1(OC(=O)CC)C(C)CC2C3CCC4=CC(=O)C=CC4(C)C3(Cl)C(O)CC12C | 0 | Train |
| 263 | OCC(O)CO | 0 | Train |
| 264 | S=C1N=CNC2=C1NC=N2 | 0 | Test |
| 265 | CCS(=O)(=O)CCN1C(C)=NC=C1[N+]([O-])=O | 0 | Train |
| 266 | OC1=CC=C(C=C1)C1C[NH2+]CCC2=C1C=C(O)C(O)=C2Cl | 0 | Train |
| 267 | CC(=O)NCCCS(O)(=O)=O | 1 | Train |
| 268 | NC(CC1=CC(I)=C(OC2=CC=C(O)C(I)=C2)C(I)=C1)C(O)=O | 0 | Train |
| 269 | CCCC[N+]1(C)C2CC(CC1C1OC21)OC(=O)C(CO)C1=CC=CC=C1 | 0 | Test |
| 270 | NC1=NC(NC2CC2)=C2N=CN([C@@H]3C[C@H](CO)C=C3)C2=N1 | 1 | Train |
| 271 | CCCCNC(=O)NS(=O)(=O)C1=CC=C(C)C=C1 | 0 | Train |
| 272 | C#CCN[C@@H]1CCC2=C1C=CC=C2 | 1 | Test |
| 273 | CO[C@@H]1C[C@@H](CC[C@H]1O)\C=C(/C)[C@H]1OC(=O)[C@@H]2CCCCN2C(=O)C(=O)[C@]2(O)O[C@@H]([C@H](C[C@H]2C)OC)[C@H](C[C@@H](C)C\C(C)=C/[C@@H](CC=C)C(=O)C[C@H](O)[C@H]1C)OC | 1 | Test |
| 274 | O=C1N(C[C@@H]2CCCC3=C2C1=CC=C3)[C@@H]1CN2CCC1CC2 | 0 | Train |
| 275 | CCC(=O)[C@@]1(C)[C@H](C)CC2C3CCC4=CC(=O)C=C[C@]4(C)C3[C@@H](O)C[C@]12C | 0 | Train |
| 276 | CC(C)C[C@H](CN)CC(O)=O | 1 | Train |
| 277 | CCCC(=O)OC1(C(C)CC2C3CCC4=CC(=O)C=CC4(C)C3(F)C(=O)CC12C)C(=O)CCl | 0 | Train |
| 278 | OCCN1CC(O)C(O)C(O)C1CO | 0 | Train |
| 279 | CNCCC(OC1=CC=C(C=C1)C(F)(F)F)C1=CC=CC=C1 | 1 | Train |
| 280 | CNS(=O)(=O)CC1=CC=C2NC=C(CCN(C)C)C2=C1 | 1 | Train |
| 281 | C\C(\C=C\C1=C(C)CCCC1(C)C)=C/C=C/C(/C)=C/C(O)=O | 1 | Train |
| 282 | CC1(O)CCC2C3CCC4=CC(=O)CCC4(C)C3(F)C(O)CC12C | 0 | Train |
| 283 | CN(C)CCCN1C2=C(CCC3=C1C=C(Cl)C=C3)C=CC=C2 | 1 | Train |
| 284 | CC(C)CC1=CC=C(C=C1)C(C)C(O)=O | 1 | Train |
| 285 | C[C@@H](C1=C(F)C=NC=N1)[C@](O)(CN1C=NC=N1)C1=CC=C(F)C=C1F | 1 | Train |
| 286 | COCCOC(=O)C1=C(C)NC(C)=C(C1C1=CC=CC(=C1)[N+]([O-])=O)C(=O)OC(C)C | 0 | Train |
| 287 | COC1=CC(OC)=C(Cl)C2=C1C(=O)C1(O2)C(C)CC(=O)C=C1OC | 0 | Test |
| 288 | NCC1CCC(CC1)C(O)=O | 1 | Train |
| 289 | CC[NH+](CC)CCOC1=CC=C(C=C1)C(=C(\Cl)C1=CC=CC=C1)\C1=CC=CC=C1 | 1 | Train |
| 290 | OCCNCCNC1=CC=C(NCCNCCO)C2=C1C(=O)C1=C(C(O)=CC=C1O)C2=O | 1 | Train |
| 291 | CC(C(O)=O)C1=CC=C(C=C1)C(=O)C1=CC=CS1 | 0 | Train |
| 292 | COC(=O)C1=C(C)NC(C)=C(C1C1=C(C=CC=C1)[N+]([O-])=O)C(=O)OCC(C)C | 0 | Train |
| 293 | C[C@H](CC1=CC=CC=C1)N(C)CC#C | 1 | Train |
| 294 | NC1=CC=CC2=C1C(=O)N(C1CCC(=O)NC1=O)C2=O | 1 | Train |
| 295 | CCN(CC)C(C)C(=O)C1=CC=CC=C1 | 0 | Train |
| 296 | O=C(O[C@H]1CN2CCC1CC2)N1CCC2=C(C=CC=C2)[C@@H]1C1=CC=CC=C1 | 1 | Train |
| 297 | CCC(=O)N(C1=CC=CC=C1)C1(CCN(CCC(=O)OC)CC1)C(=O)OC | 1 | Train |
| 298 | CCC1(CCC(=O)NC1=O)C1=CC=C(N)C=C1 | 0 | Train |
| 299 | CC1CC2C3CCC(O)(C(C)=O)C3(C)CC(O)C2(F)C2(C)C=CC(=O)C=C12 | 0 | Train |
| 300 | COC1=CC=C(CCN(C)CCCC(C#N)(C(C)C)C2=CC=C(OC)C(OC)=C2)C=C1OC | 0 | Train |
| 301 | CC(C)NCC(O)COC1=C(OCC=C)C=CC=C1 | 0 | Train |
| 302 | O[C@@H]1CO[C@@H](O[C@@H]2CO[C@@H](O)[C@H](OS(O)(=O)=O)[C@H]2OS(O)(=O)=O)[C@H](OS(O)(=O)=O)[C@H]1OS(O)(=O)=O | 0 | Train |
| 303 | CC(=O)[C@@]1(O)CC[C@H]2[C@@H]3CCC4=CC(=O)CC[C@]4(C)[C@H]3CC[C@]12C | 0 | Train |
| 304 | CNC1(CCCCC1=O)C1=C(Cl)C=CC=C1 | 0 | Train |
| 305 | NC1=C(F)C=NC(=O)N1 | 0 | Test |
| 306 | OC(=O)C1=C(O)C=CC=C1 | 0 | Train |
| 307 | CC[C@H]1OC(=O)[C@H](C)[C@@H](O[C@H]2C[C@@](C)(OC)[C@@H](O)[C@H](C)O2)[C@H](C)[C@@H](O[C@@H]2O[C@H](C)C[C@@H]([C@H]2O)N(C)C)[C@@](C)(C[C@@H](C)C(=O)[C@H](C)[C@@H](O)[C@]1(C)O)OC | 1 | Train |
| 308 | CC[C@H]1OC(=O)[C@H](C)[C@@H](O[C@H]2C[C@@](C)(OC)[C@@H](O)[C@H](C)O2)[C@H](C)[C@@H](O[C@@H]2O[C@H](C)C[C@@H]([C@H]2O)N(C)C)[C@](C)(O)C[C@@H](C)CN(C)[C@H](C)[C@@H](O)[C@]1(C)O | 1 | Train |
| 309 | CC(C)C1=C(C(=O)NC2=CC=CC=C2)C(=C(N1CC[C@@H](O)C[C@@H](O)CC([O-])=O)C1=CC=C(F)C=C1)C1=CC=CC=C1 | 1 | Train |
| 310 | COC1=C(OCC2CCN(C)CC2)C=C2N=CN=C(NC3=CC=C(Br)C=C3F)C2=C1 | 1 | Train |
| 311 | CCCCCCCCCCCCCCCCCCCCCCO | 0 | Test |
| 312 | OCC1OC(CC1O)N1C=C(I)C(=O)NC1=O | 0 | Train |
| 313 | CC1(C)SC2C(NC(=O)C(N)C3=CC=CC=C3)C(=O)N2C1C([O-])=O | 0 | Train |
| 314 | CC(=O)O[C@H]1[C@H](CC2C3CCC4C[C@H](O)[C@H](C[C@]4(C)C3CC[C@]12C)N1CCOCC1)[N+]1(CC=C)CCCC1 | 0 | Train |
| 315 | [O-]C(=O)C1=CC=CN=C1 | 0 | Test |
| 316 | CC1=C(SC=C1)C(=CCCN1CCC[C@H](C1)C(O)=O)C1=C(C)C=CS1 | 1 | Train |
| 317 | NC1=NN=C(C(N)=N1)C1=CC=CC(Cl)=C1Cl | 1 | Train |
| 318 | CC1=NC2=C(CCN(C(=O)C3=CC=C(NC(=O)C4=C(C=CC=C4)C4=CC=CC=C4)C=C3)C3=C2C=CC=C3)N1 | 0 | Train |
| 319 | C(C(C1CCCCC1)C1CCCCC1)C1CCCCN1 | 0 | Train |
| 320 | CCN(CC)CCOC(=O)C1=CC=C(N)C=C1Cl | 0 | Train |
| 321 | CCN(CCCCOC(=O)C1=CC=C(OC)C(OC)=C1)C(C)CC1=CC=C(OC)C=C1 | 0 | Train |
| 322 | CC(C)C1=CC=C2OC3=C(C=C(C(O)=O)C(N)=N3)C(=O)C2=C1 | 0 | Train |
| 323 | [O-][N+](=O)OCCNC(=O)C1=CC=CN=C1 | 0 | Train |
| 324 | CCC1=C2CN3C(=CC4=C(COC(=O)[C@]4(O)CC)C3=O)C2=NC2=CC=C(OC(=O)N3CCC(CC3)N3CCCCC3)C=C12 | 1 | Train |
| 325 | CN(CC=CC#CC(C)(C)C)CC1=C2C=CC=CC2=CC=C1 | 0 | Train |
| 326 | CC(C)(C)NCC(O)COC1=C2CC(O)C(O)CC2=CC=C1 | 0 | Train |
| 327 | NC(=O)N[C@@H](CCC(O)=O)C(O)=O | 0 | Train |
| 328 | NC1=NC(=O)N(C=C1)[C@@H]1O[C@H](CO)[C@@H](O)C1(F)F | 1 | Train |
| 329 | NC1=NC(=O)N(C=C1F)[C@@H]1CS[C@H](CO)O1 | 1 | Test |
| 330 | CC(CCC(O)=O)C1CCC2C3C(O)CC4CC(O)CCC4(C)C3CC(O)C12C | 0 | Train |
| 331 | CO[C@@H]1C[C@H](C[C@@H](C)[C@@H]2CC(=O)[C@H](C)\C=C(C)/[C@@H](O)[C@@H](OC)C(=O)[C@H](C)C[C@H](C)\C=C\C=C\C=C(C)\[C@H](C[C@@H]3CC[C@@H](C)[C@@](O)(O3)C(=O)C(=O)N3CCCC[C@H]3C(=O)O2)OC)CC[C@H]1OCCO | 1 | Train |
| 332 | CC(O)C(O)=O | 0 | Train |
| 333 | NCCCC[C@H](NC(=O)[C@@H]1CCCN1C(=O)[C@@H]1CSSC[C@H](NC(=O)CNC(=O)CNC(=O)CN)C(=O)N[C@@H](CC2=CC=C(O)C=C2)C(=O)N[C@@H](CC2=CC=CC=C2)C(=O)N[C@@H](CCC(N)=O)C(=O)N[C@@H](CC(N)=O)C(=O)N1)C(=O)NCC(N)=O | 0 | Train |
| 334 | O=C1NC(=O)C(N1)(C1=CC=CC=C1)C1=CC=CC=C1 | 1 | Test |
| 335 | NCCCC[C@H](N[C@@H](CCC1=CC=CC=C1)C(O)=O)C(=O)N1CCC[C@H]1C(O)=O | 1 | Train |
| 336 | CC1=NC=C(N=C1)C(=O)NCCC1=CC=C(C=C1)S(=O)(=O)NC(=O)NC1CCCCC1 | 0 | Train |
| 337 | CC(C)(C)NCC(O)COC1=C2CCC(=O)NC2=CC=C1 | 0 | Train |
| 338 | CCCCCN(C)CCC(O)(P(O)(O)=O)P(O)(O)=O | 1 | Train |
| 339 | CN(C)C\C=C\C(=O)NC1=C(O[C@H]2CCOC2)C=C2N=CN=C(NC3=CC=C(F)C(Cl)=C3)C2=C1 | 1 | Train |
| 340 | CN1C2CC(CC1C1OC21)OC(=O)C(CO)C1=CC=CC=C1 | 0 | Train |
| 341 | CCCN1C[C@H](CSC)C[C@H]2[C@H]1CC1=CNC3=CC=CC2=C13 | 1 | Train |
| 342 | CC#CC1(O)CCC2C3CCC4=CC(=O)CCC4=C3C(CC12C)C1=CC=C(C=C1)N(C)C | 0 | Train |
| 343 | CC(N)(CC1=CC=C(O)C=C1)C(O)=O | 0 | Train |
| 344 | CC[N+](C)(C)CC1=C(Br)C=CC=C1 | 0 | Train |
| 345 | COC1=C(OCC(O)COC(N)=O)C=CC=C1 | 0 | Train |
| 346 | COC(=O)C1=C(C)NC(C)=C(C1C1=CC=CC(=C1)[N+]([O-])=O)C(=O)OCC[NH+](C)CC1=CC=CC=C1 | 0 | Train |
| 347 | NC(N)=NC(=O)C1=NC(Cl)=C(N)N=C1N | 0 | Test |
| 348 | NC1=NC2=C(C(CCC3=CC=C(C=C3)C(=O)N[C@@H](CCC(O)=O)C(O)=O)=CN2)C(=O)N1 | 1 | Train |
| 349 | O=C1[C@H]2[C@@H]3CC[C@@H](C3)[C@H]2C(=O)N1C[C@@H]1CCCC[C@H]1CN1CCN(CC1)C1=NSC2=CC=CC=C12 | 1 | Train |
| 350 | CCOC(=O)C1=C(C)NC(C)=C(C1C1=CC=CC(Cl)=C1Cl)C(=O)OC | 0 | Train |
| 351 | NC1=NC(=O)N(C[C@@H](CO)OCP(O)(O)=O)C=C1 | 1 | Train |
| 352 | CC(C)C[C@H](NC(=O)[C@H](CC1=CC=CC=C1)NC(=O)C1=CN=CC=N1)B(O)O | 1 | Test |
| 353 | CO[C@@H]1C[C@H](C[C@@H](C)[C@@H]2CC(=O)[C@H](C)\C=C(C)/[C@@H](O)[C@@H](OC)C(=O)[C@H](C)C[C@H](C)\C=C\C=C\C=C(C)\[C@H](C[C@@H]3CC[C@@H](C)[C@@](O)(O3)C(=O)C(=O)N3CCCC[C@H]3C(=O)O2)OC)CC[C@H]1O | 1 | Train |
| 354 | CCCCNC1=CC=C(C=C1)C(=O)OCCN(C)C | 0 | Train |
| 355 | CC1=CC=C(C=C1)C(=O)C1=CC(O)=C(O)C(=C1)[N+]([O-])=O | 1 | Train |
| 356 | CCC1=NN(CCCN2CCN(CC2)C2=CC=CC(Cl)=C2)C(=O)N1CCOC1=CC=CC=C1 | 1 | Train |
| 357 | CC1=NC(=CC(NC2=NC=C(S2)C(=O)NC2=C(Cl)C=CC=C2C)=N1)N1CCN(CCO)CC1 | 1 | Train |
| 358 | [O-]C(=O)COCC[NH+]1CC[NH+](CC1)[C@H](C1=CC=CC=C1)C1=CC=C(Cl)C=C1 | 1 | Train |
| 359 | OC(=O)CCNC(=O)C1=CC=C(C=C1)N=NC1=CC=C(O)C(=C1)C(O)=O | 1 | Train |
| 360 | COC1=CC=C(C=C1)C(CN(C)C)C1(O)CCCCC1 | 1 | Train |
| 361 | CC(COC1=CC=CC=C1)N(CCCl)CC1=CC=CC=C1 | 0 | Train |
| 362 | OC(CNCC(O)C1CCC2=C(O1)C=CC(F)=C2)C1CCC2=C(O1)C=CC(F)=C2 | 1 | Train |
| 363 | CCC(C)C(N)C1=NC(CS1)C(=O)NC(CC(C)C)C(=O)NC(CCC(O)=O)C(=O)NC(C(C)CC)C(=O)NC1CCCCNC(=O)C(CC(N)=O)NC(=O)C(CC(O)=O)NC(=O)C(CC2=CN=CN2)NC(=O)C(CC2=CC=CC=C2)NC(=O)C(NC(=O)C(CCCN)NC1=O)C(C)CC | 0 | Train |
| 364 | CCC(C)(C)C(=O)O[C@H]1C[C@@H](C)C=C2C=C[C@H](C)[C@H](CC[C@@H]3C[C@@H](O)CC(=O)O3)[C@@H]12 | 1 | Train |
| 365 | ClCCNP1(=O)OCCCN1CCCl | 1 | Train |
| 366 | CC1=C(CS(=O)C2=NC3=CC=CC=C3N2)N=CC=C1OCC(F)(F)F | 1 | Train |
| 367 | CC(C)C(C)C=CC(C)C1CCC2C(CCCC12C)=CC=C1CC(O)CCC1=C | 0 | Train |
| 368 | CC(C)CCCC(C)CCCC(C)CCCC(C)=CCC1=C(C)C(=O)C2=C(C=CC=C2)C1=O | 0 | Train |
| 369 | NS(=O)(=O)CC1=NOC2=CC=CC=C12 | 1 | Test |
| 370 | CN(C)CCOC(C1=CC=CC=C1)C1=CC=CC=C1 | 0 | Train |
| 371 | CC(C)(C)C(=O)OCOP(=O)(COCCN1C=NC2=C(N)N=CN=C12)OCOC(=O)C(C)(C)C | 1 | Train |
| 372 | CCN(CC)CCOC(=O)C1(CCCCC1)C1CCCCC1 | 0 | Test |
| 373 | CN1C=CC(=O)C(O)=C1C | 1 | Train |
| 374 | OC(C(=O)OC1CC2CC[C@@H](C1)[N+]21CCCC1)(C1=CC=CC=C1)C1=CC=CC=C1 | 1 | Train |
| 375 | CC(=O)O[C@@]12CO[C@@H]1C[C@H](O)[C@]1(C)[C@@H]2[C@H](OC(=O)C2=CC=CC=C2)[C@]2(O)C[C@H](OC(=O)C(O)[C@@H](NC(=O)OC(C)(C)C)C3=CC=CC=C3)C(C)=C([C@@H](O)C1=O)C2(C)C | 1 | Test |
| 376 | CNCC[C@H](OC1=C2C=CC=CC2=CC=C1)C1=CC=CS1 | 1 | Train |
| 377 | CN1CCN(CC1)C1=NC2=C(NC3=C1C=C(C)S3)C=CC=C2 | 1 | Train |
| 378 | CC[C@H]1[C@@H](O)[C@H]2[C@@H]3CC[C@H]([C@H](C)CCC(O)=O)[C@@]3(C)CC[C@@H]2[C@@]2(C)CC[C@@H](O)C[C@@H]12 | 0 | Train |
| 379 | NC1=NC(=CS1)C(=N\O)\C(=O)N[C@H]1[C@H]2SCC(C=C)=C(N2C1=O)C(O)=O | 1 | Train |
| 380 | C[C@]12C[C@H](O)[C@@]3(F)[C@@H](CCC4=CC(=O)CC[C@]34C)[C@@H]1CC[C@]2(O)C(=O)CO | 0 | Train |
| 381 | CC(C)(C)C(O)C=CC1=CC=C2OCOC2=C1 | 0 | Train |
| 382 | CCC(C)CC(C)CCCCCCCCC(=O)N[C@H]1C[C@@H](O)[C@@H](NCCN)NC(=O)[C@@H]2[C@@H](O)CCN2C(=O)[C@@H](NC(=O)[C@@H](NC(=O)[C@@H]2C[C@@H](O)CN2C(=O)[C@@H](NC1=O)[C@@H](C)O)[C@H](O)[C@@H](O)C1=CC=C(O)C=C1)[C@H](O)CCN | 1 | Train |
| 383 | COC(=O)[C@@H]1CC2=CC(=O)CC[C@]2(C)[C@@]23O[C@@H]2C[C@@]2(C)[C@@H](CC[C@@]22CCC(=O)O2)[C@H]13 | 1 | Train |
| 384 | OC(=O)C1=CC(=CC=C1O)N=NC1=CC=C(C=C1)S(=O)(=O)NC1=NC=CC=C1 | 1 | Test |
| 385 | CC(CO)NC(=O)C1CN(C)C2CC3=CNC4=CC=CC(=C34)C2=C1 | 0 | Train |
| 386 | CC(C)N1C(\C=C\[C@@H](O)C[C@@H](O)CC(O)=O)=C(C2=CC=CC=C12)C1=CC=C(F)C=C1 | 1 | Train |
| 387 | ClC1=CC(=CC=C1)N1CCN(CCCN2N=C3C=CC=CN3C2=O)CC1 | 0 | Train |
| 388 | CC(=O)OC1=CC=C(C=C1)C(C1=CC=C(OC(C)=O)C=C1)C1=NC=CC=C1 | 0 | Train |
| 389 | CN1C(=O)C([O-])=C(N=C1C(C)(C)NC(=O)C1=NN=C(C)O1)C(=O)NCC1=CC=C(F)C=C1 | 1 | Test |
| 390 | COC1=CC(Br)=C(C[N+]2(CCOCCC3CCC4CC3C4(C)C)CCOCC2)C=C1OC | 0 | Train |
| 391 | [O-]C(=O)CN(CCN(CC([O-])=O)CC([O-])=O)CCN(CC([O-])=O)CC([O-])=O | 1 | Test |
| 392 | CC(C)NCC(O)COC1=CC=C(CC(N)=O)C=C1 | 1 | Train |
| 393 | C[C@H]1COC2=C(N3CCN(C)CC3)C(F)=CC3=C2N1C=C(C(O)=O)C3=O | 1 | Train |
| 394 | CC(C)CN(C[C@@H](O)[C@H](CC1=CC=CC=C1)NC(=O)O[C@H]1CO[C@H]2OCC[C@@H]12)S(=O)(=O)C1=CC=C(N)C=C1 | 1 | Test |
| 395 | CO\N=C(/C(=O)N[C@H]1[C@H]2SCC(COC(C)=O)=C(N2C1=O)C(O)=O)C1=CSC(N)=N1 | 1 | Train |
| 396 | CCCC1=NC2=C(C)C=C(C=C2N1CC1=CC=C(C=C1)C1=C(C=CC=C1)C(O)=O)C1=NC2=CC=CC=C2N1C | 1 | Train |
| 397 | CN(CC1=CC=C(C=C1)C(C)(C)C)CC1=C2C=CC=CC2=CC=C1 | 0 | Train |
| 398 | OC(CN1C=CN=C1)(P(O)(O)=O)P(O)(O)=O | 1 | Train |
| 399 | CCCCCCOC(=O)N=C(N)C1=CC=C(NCC2=NC3=CC(=CC=C3N2C)C(=O)N(CCC(=O)OCC)C2=NC=CC=C2)C=C1 | 0 | Train |
| 400 | OC(=O)C1=CN(C2CC2)C2=C(C=C(F)C(=C2)N2CCNCC2)C1=O | 1 | Train |
| 401 | CCC(=O)O[C@@](CC1=CC=CC=C1)([C@H](C)CN(C)C)C1=CC=CC=C1 | 0 | Train |
| 402 | N[C@@H](CC1=CC=C(O)C=C1)C(O)=O | 1 | Train |
| 403 | NC[C@@H]1O[C@H](O[C@@H]2[C@@H](CO)O[C@@H](O[C@@H]3[C@@H](O)[C@H](N)C[C@H](N)[C@H]3O[C@H]3O[C@H](CN)[C@@H](O)[C@H](O)[C@H]3N)[C@@H]2O)[C@H](N)[C@@H](O)[C@@H]1O | 0 | Train |
| 404 | NCC(O)C1=CC=C(O)C(O)=C1 | 0 | Train |
| 405 | COCCCOC1=CC=NC(CS(=O)C2=NC3=CC=CC=C3N2)=C1C | 1 | Test |
| 406 | CCCC(=O)OC1(CCC2C3CC(F)C4=CC(=O)C=CC4(C)C3(F)C(O)CC12C)C(=O)COC(C)=O | 0 | Train |
| 407 | CCCNC(=O)NS(=O)(=O)C1=CC=C(Cl)C=C1 | 0 | Train |
| 408 | CO\N=C(/C(=O)N[C@H]1[C@H]2SCC(\C=C/C3=C(C)N=CS3)=C(N2C1=O)C(=O)OCOC(=O)C(C)(C)C)C1=CSC(N)=N1 | 1 | Test |
| 409 | CCOC(=O)C1=CC=C(N)C=C1 | 0 | Test |
| 410 | CC1=C(C[S@@](=O)C2=NC3=CC=CC=C3N2)N=CC=C1OCC(F)(F)F | 1 | Train |
| 411 | FC(F)(F)[C@]1(OC(=O)NC2=C1C=C(Cl)C=C2)C#CC1CC1 | 1 | Train |
| 412 | COCCCOC1=CC(C[C@@H](C[C@H](N)[C@@H](O)C[C@@H](C(C)C)C(=O)NCC(C)(C)C(N)=O)C(C)C)=CC=C1OC | 1 | Train |
| 413 | CCC1=C(C)NC2=C1C(=O)C(CN1CCOCC1)CC2 | 0 | Train |
| 414 | FC1=CC=C(C=C1)[C@@H]1CCNC[C@H]1COC1=CC=C2OCOC2=C1 | 1 | Train |
| 415 | CO[C@H]1C=CO[C@@]2(C)OC3=C(C)C(O)=C4C(O)=C(NC(=O)C(C)=CC=C[C@H](C)[C@H](O)[C@@H](C)[C@@H](O)[C@@H](C)[C@H](OC(C)=O)[C@@H]1C)C1=C(N=C5C=C(C)C=CN15)C4=C3C2=O | 0 | Train |
| 416 | CC1=C(N=NC2=C(O)C(=CC=C2)C2=CC=CC(=C2)C(O)=O)C(=O)N(N1)C1=CC=C(C)C(C)=C1 | 0 | Train |
| 417 | CCCC(=O)OCOC(=O)C1=C(C)NC(C)=C(C1C1=CC=CC(Cl)=C1Cl)C(=O)OC | 1 | Train |
| 418 | CCC[C@@]1(CCC2=CC=CC=C2)CC([O-])=C([C@H](CC)C2=CC=CC([N-]S(=O)(=O)C3=CC=C(C=N3)C(F)(F)F)=C2)C(=O)O1 | 1 | Train |
| 419 | CN[C@@H]1CCC2=C(C1)C1=CC(=CC=C1N2)C(N)=O | 1 | Test |
| 420 | C[C@H]1CN(C[C@H](CC2=CC=CC=C2)C(=O)NCC(O)=O)CC[C@@]1(C)C1=CC=CC(O)=C1 | 0 | Train |
| 421 | CC(C)NCC(O)COC1=C2C=CC=CC2=CC=C1 | 0 | Test |
| 422 | NC1=NC(=CS1)C(=N/OCC(O)=O)\C(=O)N[C@H]1[C@H]2SCC(C=C)=C(N2C1=O)C(O)=O | 1 | Train |
| 423 | CC1(C)S[C@@H]2[C@H](NC(=O)CC3=CC=CC=C3)C(=O)N2[C@H]1C(O)=O | 0 | Train |
| 424 | CC(C=CC(O)C1CC1)C1CCC2C(CCCC12C)=CC=C1CC(O)CC(O)C1=C | 0 | Train |
| 425 | O[C@H]1CO[C@@H]2[C@@H](CO[C@H]12)O[N+]([O-])=O | 0 | Test |
| 426 | CO[C@@H]1C[C@H](C[C@@H](C)[C@@H]2CC(=O)[C@H](C)\C=C(C)/[C@@H](O)[C@@H](OC)C(=O)[C@H](C)C[C@H](C)\C=C\C=C\C=C(C)\[C@H](C[C@@H]3CC[C@@H](C)[C@@](O)(O3)C(=O)C(=O)N3CCCC[C@H]3C(=O)O2)OC)CC[C@H]1OC(=O)C(C)(CO)CO | 1 | Train |
| 427 | C[C@H](\C=C\[C@H](C)C(C)(C)O)[C@H]1CC[C@H]2\C(CCC[C@]12C)=C\C=C1C[C@@H](O)C[C@H](O)C1 | 1 | Train |
| 428 | CN(C)CCC1=CNC2=CC=C(C[C@H]3COC(=O)N3)C=C12 | 1 | Train |
| 429 | C[N+]1(CC2CC2)CC[C@@]23[C@H]4OC5=C2C(C[C@@H]1[C@]3(O)CCC4=O)=CC=C5O | 0 | Train |
| 430 | CC(CN1C2=C(SC3=C1C=CC=C3)C=CC=C2)N(C)C | 0 | Train |
| 431 | CC(C)COC1=CC=C(C=C1C#N)C1=NC(C)=C(S1)C(O)=O | 1 | Train |
| 432 | NC1=CC=CC2=C1CN(C1CCC(=O)NC1=O)C2=O | 1 | Test |
| 433 | CC1=CN([C@H]2C[C@H](N=[N+]=[N-])[C@@H](CO)O2)C(=O)NC1=O | 1 | Train |
| 434 | CC(O)C(CO)NC(=O)C1CSSCC(NC(=O)C(N)CC2=CC=CC=C2)C(=O)NC(CC2=CC=CC=C2)C(=O)NC(CC2=CNC3=CC=CC=C23)C(=O)NC(CCCCN)C(=O)NC(C(C)O)C(=O)N1 | 1 | Train |
| 435 | COC1=C(OCCNCC(O)COC2=CC=CC3=C2C2=C(N3)C=CC=C2)C=CC=C1 | 1 | Train |
| 436 | CN(C)C(=O)CC1=C(N=C2C=CC(C)=CN12)C1=CC=C(C)C=C1 | 1 | Train |
| 437 | CC(=O)N(O)CCCCCNC(=O)CCC(=O)N(O)CCCCCNC(=O)CCC(=O)N(O)CCCCCN | 1 | Train |
| 438 | NC1=CC=C(O)C(=C1)C(O)=O | 1 | Train |
| 439 | NCC1(CC(O)=O)CCCCC1 | 1 | Train |
| 440 | NC(N)=NC(=O)CC1=C(Cl)C=CC=C1Cl | 1 | Train |
| 441 | NC(CCC(O)=O)C=C | 1 | Train |
| 442 | CC12CC3CC(C)(C1)CC(N)(C3)C2 | 1 | Train |
| 443 | CCCN(CCC)CCC1=CC=CC2=C1CC(=O)N2 | 1 | Train |
| 444 | CC(C)OC(=O)C(C)(C)OC1=CC=C(C=C1)C(=O)C1=CC=C(Cl)C=C1 | 1 | Train |
| 445 | OCC(NC(=O)C(Cl)Cl)C(O)C1=CC=C(C=C1)[N+]([O-])=O | 1 | Train |
| 446 | CC[C@@H]1C=C(C)C[C@H](C)C[C@H](OC)[C@H]2O[C@](O)([C@H](C)C[C@@H]2OC)C(=O)C(=O)N2CCCC[C@H]2C(=O)O[C@@H]([C@H](C)[C@@H](O)CC1=O)C(C)=C[C@@H]1CC[C@H](Cl)[C@@H](C1)OC | 0 | Train |
| 447 | NS(=O)(=O)C1=CC2=C(NC(CC3=CC=CC=C3)NS2(=O)=O)C=C1C(F)(F)F | 0 | Test |
| 448 | CN(C)CCCOC1=NN(CC2=CC=CC=C2)C2=CC=CC=C12 | 0 | Test |
| 449 | NC1=C2N=CN(C3OC(CO)C(O)C3O)C2=NC=N1 | 0 | Train |
| 450 | CC(C)N(CC[C@H](C1=CC=CC=C1)C1=CC(CO)=CC=C1OC(=O)C(C)C)C(C)C | 0 | Test |
| 451 | NCCNCCNCCNCCN | 0 | Train |
| 452 | O=C(OOC(=O)C1=CC=CC=C1)C1=CC=CC=C1 | 0 | Train |
| 453 | CCOC(=O)C1=CN=CN1C(C)C1=CC=CC=C1 | 0 | Train |
| 454 | CC(CCC1=CC=C(O)C=C1)NCCC1=CC=C(O)C(O)=C1 | 0 | Train |
| 455 | CN1CCC2=CC=CC3=C2C1CC1=C3C(O)=C(O)C=C1 | 0 | Test |
| 456 | CCN(CC)CC(=O)NC1=C(C)C=CC=C1C | 0 | Test |
| 457 | CN(C)C1=CC=C(O)C2=C1C[C@H]1C[C@H]3[C@H]([NH+](C)C)C(=O)C(C(N)=O)=C([O-])[C@@]3(O)C(=O)C1=C2[O-] | 1 | Train |
| 458 | CN[C@H]1CC[C@@H](C2=CC=C(Cl)C(Cl)=C2)C2=C1C=CC=C2 | 1 | Train |
| 459 | OC(=O)C1CCN2C1=CC=C2C(=O)C1=CC=CC=C1 | 1 | Train |
| 460 | CN[C@H](CC(C)C)C(=O)N[C@@H]1[C@H](O)C2=CC=C(OC3=C(O[C@@H]4O[C@H](CO)[C@@H](O)[C@H](O)[C@H]4O[C@H]4C[C@](C)(N)[C@H](O)[C@H](C)O4)C4=CC(=C3)[C@@H](NC(=O)[C@H](CC(N)=O)NC1=O)C(=O)N[C@@H]1C3=CC=C(O)C(=C3)C3=C(C=C(O)C=C3O)[C@H](NC(=O)[C@@H](NC1=O)[C@H](O)C1=CC(Cl)=C(O4)C=C1)C(O)=O)C(Cl)=C2 | 1 | Train |
| 461 | CN1C2=C(NC=N2)C(=O)N(C)C1=O | 1 | Train |
| 462 | COC1=CC(=CC=C1)[C@@]1(O)CCCC[C@@H]1CN(C)C | 1 | Train |
| 463 | CNC(=O)C1=CC(OC2=CC=C(NC(=O)NC3=CC=C(Cl)C(=C3)C(F)(F)F)C=C2)=CC=N1 | 1 | Train |
| 464 | CC1=CN([C@@H]2C[C@@H](O)[C@H](CO)O2)C(=O)NC1=O | 1 | Test |
| 465 | OC(=O)COCCN1CCN(CC1)C(C1=CC=CC=C1)C1=CC=C(Cl)C=C1 | 1 | Train |
| 466 | NC(=O)C1=NN(C=N1)[C@@H]1O[C@H](CO)[C@@H](O)[C@H]1O | 1 | Train |
| 467 | NCCC(O)(P(O)([O-])=O)P(O)([O-])=O | 1 | Train |
| 468 | C[C@H]1O[C@@H](O[C@@H]2C[C@@H]3O[C@@](O)(C[C@H](O)[C@H]3C(O)=O)C[C@@H](O)C[C@@H](O)[C@H](O)CC[C@@H](O)C[C@@H](O)CC(=O)O[C@@H](C)[C@H](C)[C@H](O)[C@@H](C)\C=C\C=C\C=C\C=C\C=C\C=C\C=C\2)[C@@H](O)[C@@H](N)[C@@H]1O | 1 | Train |
| 469 | COC1=CC2=CC=C(CCC(C)=O)C=C2C=C1 | 1 | Train |
| 470 | CC(C)CN(C[C@@H](O)[C@H](CC1=CC=CC=C1)NC(=O)O[C@H]1CCOC1)S(=O)(=O)C1=CC=C(N)C=C1 | 1 | Train |
| 471 | COCCOC1=C(OCCOC)C=C2C(NC3=CC(=CC=C3)C#C)=NC=NC2=C1 | 1 | Train |
| 472 | OC[C@H]1O[C@H]([C@H](O)[C@@H](O)[C@@H]1O)C1=CC=C(Cl)C(CC2=CC=C(O[C@H]3CCOC3)C=C2)=C1 | 1 | Train |
| 473 | CC1=CN(C=N1)C1=CC(NC(=O)C2=CC=C(C)C(NC3=NC(=CC=N3)C3=CC=CN=C3)=C2)=CC(=C1)C(F)(F)F | 1 | Train |
| 474 | OC(=O)CCCC1=CC=CC=C1 | 1 | Train |
| 475 | OC(CN1C=NC=N1)(CN1C=NC=N1)C1=CC=C(F)C=C1F | 1 | Train |
| 476 | CC(C)(O\N=C(\C(=O)N[C@H]1[C@H]2SCC(C[N+]3=CC=CC=C3)=C(N2C1=O)C([O-])=O)C1=CSC(N)=N1)C(O)=O | 1 | Train |
| 477 | CCC[C@H](N[C@H](C)C(=O)N1[C@H]2CCCC[C@H]2C[C@H]1C(O)=O)C(=O)OCC | 1 | Train |
| 478 | C[C@H](N[C@@H](CCC1=CC=CC=C1)C(O)=O)C(=O)N1[C@H]2CCCC[C@@H]2C[C@H]1C(O)=O | 1 | Train |
| 479 | CC[C@H](C)[C@H](NC(=O)[C@H](CC1=CC=CC=C1)NC(=O)[C@H](CC(C)C)NC(=O)[C@H](CCCNC(N)=N)NC(=O)[C@@H](NC(=O)[C@H](C)NC(=O)[C@H](CCC(O)=O)NC(=O)[C@H](CCC(O)=O)NC(=O)[C@H](CCC(O)=O)NC(=O)[C@H](CCSC)NC(=O)[C@H](CCC(N)=O)NC(=O)[C@H](CCCCN)NC(=O)[C@H](CO)NC(=O)[C@H](CC(C)C)NC(=O)[C@H](CC(O)=O)NC(=O)[C@H](CO)NC(=O)[C@@H](NC(=O)[C@H](CC1=CC=CC=C1)NC(=O)[C@@H](NC(=O)CNC(=O)[C@H](CCC(O)=O)NC(=O)CNC(=O)[C@@H](N)CC1=CN=CN1)[C@@H](C)O)[C@@H](C)O)C(C)C)C(=O)N[C@@H](CCC(O)=O)C(=O)N[C@@H](CC1=CNC2=CC=CC=C12)C(=O)N[C@@H](CC(C)C)C(=O)N[C@@H](CCCCN)C(=O)N[C@@H](CC(N)=O)C(=O)NCC(=O)NCC(=O)N1CCC[C@H]1C(=O)N[C@@H](CO)C(=O)N[C@@H](CO)C(=O)NCC(=O)N[C@@H](C)C(=O)N1CCC[C@H]1C(=O)N1CCC[C@H]1C(=O)N1CCC[C@H]1C(=O)N[C@@H](CO)C(N)=O | 1 | Train |
| 480 | CN1\C(=C(/[O-])NC2=NC=CC=C2)C(=O)C2=C(C=CC=C2)S1(=O)=O | 1 | Train |
| 481 | CC(C)C[C@H](NC(=O)[C@H](CCC1=CC=CC=C1)NC(=O)CN1CCOCC1)C(=O)N[C@@H](CC1=CC=CC=C1)C(=O)N[C@@H](CC(C)C)C(=O)[C@@]1(C)CO1 | 1 | Train |
| 482 | [O-]P([O-])(=O)OCN1C(=O)NC(C1=O)(C1=CC=CC=C1)C1=CC=CC=C1 | 1 | Train |
| 483 | CCCCCCCCCCCCCCCC(=O)N[C@@H](CCC(=O)NCCCC[C@H](NC(=O)[C@H](C)NC(=O)[C@H](C)NC(=O)[C@H](CCC(N)=O)NC(=O)CNC(=O)[C@H](CCC(O)=O)NC(=O)[C@H](CC(C)C)NC(=O)[C@H](CC1=CC=C(O)C=C1)NC(=O)[C@H](CO)NC(=O)[C@H](CO)NC(=O)[C@@H](NC(=O)[C@H](CC(O)=O)NC(=O)[C@H](CO)NC(=O)[C@@H](NC(=O)[C@H](CC1=CC=CC=C1)NC(=O)[C@@H](NC(=O)CNC(=O)[C@H](CCC(O)=O)NC(=O)[C@H](C)NC(=O)[C@@H](N)CC1=CN=CN1)[C@@H](C)O)[C@@H](C)O)C(C)C)C(=O)N[C@@H](CCC(O)=O)C(=O)N[C@@H](CC1=CC=CC=C1)C(=O)N[C@@H]([C@@H](C)CC)C(=O)N[C@@H](C)C(=O)N[C@@H](CC1=CNC2=CC=CC=C12)C(=O)N[C@@H](CC(C)C)C(=O)N[C@@H](C(C)C)C(=O)N[C@@H](CCCNC(N)=N)C(=O)NCC(=O)N[C@@H](CCCNC(N)=N)C(=O)NCC(O)=O)C(O)=O | 1 | Train |
| 484 | CN1CCC[C@@H]1CC1=CNC2=CC=C(CCS(=O)(=O)C3=CC=CC=C3)C=C12 | 1 | Train |
| 485 | CC1=CC=NC2=C1NC(=O)C1=C(N=CC=C1)N2C1CC1 | 1 | Train |
| 486 | C[C@@H](OC1=CC(=CN=C1N)C1=CN(N=C1)C1CCNCC1)C1=C(Cl)C(F)=CC=C1Cl | 1 | Train |
| 487 | CC(C)NC1=CC=CN=C1N1CCN(CC1)C(=O)C1=CC2=CC(NS(C)(=O)=O)=CC=C2N1 | 1 | Train |
| 488 | CCN(C(C)=O)C1=CC(=CC=C1)C1=CC=NC2=C(C=NN12)C#N | 1 | Train |
| 489 | FC(F)(F)C(F)(F)C(F)(F)F | 1 | Train |
| 490 | CC1OC(O[C@@H]2C[C@@H]3O[C@@](O)(C[C@H](O)[C@H]3C(O)=O)C[C@@H](O)C[C@@H](O)[C@H](O)CC[C@@H](O)C[C@@H](O)CC(=O)O[C@@H](C)[C@H](C)[C@H](O)[C@@H](C)\C=C\C=C\C=C\C=C\C=C\C=C\C=C\2)C(O)C(N)C1O | 1 | Train |
| 491 | CN1CCN(CC1)C1=NC2=C(NC3=C1C=CC=C3)C=CC(Cl)=C2 | 1 | Test |
| 492 | NC1=NC2=C(N=CN2COC(CO)CO)C(=O)N1 | 1 | Train |
| 493 | C[C@H](N[C@@H](CCC1=CC=CC=C1)C(O)=O)C(=O)N1[C@H]2CCC[C@H]2C[C@H]1C(O)=O | 1 | Train |
| 494 | CN(C)CCCC1(OCC2=C1C=CC(=C2)C#N)C1=CC=C(F)C=C1 | 1 | Train |
| 495 | NC(=O)C1=CN(CC2=C(F)C=CC=C2F)N=N1 | 1 | Train |
| 496 | CCOC(=O)[C@H](CCC1=CC=CC=C1)N[C@H]1CCCN2CCC[C@H](N2C1=O)C(O)=O | 1 | Train |
| 497 | C[C@H](CN1C=NC2=C(N)N=CN=C12)OCP(O)(O)=O | 1 | Train |
| 498 | NC1=C2N=CN([C@@H]3O[C@H](CO)[C@@H](O)[C@@H]3O)C2=NC(F)=N1 | 1 | Test |
| 499 | COC1=C(N2C[C@@H]3CCCN[C@@H]3C2)C(F)=CC2=C1N(C=C(C(O)=O)C2=O)C1CC1 | 1 | Test |
| 500 | [O-]C(=O)CN(CC([O-])=O)C1=C(C#N)C(CC([O-])=O)=C(S1)C([O-])=O | 0 | Test |
| 501 | CCOC1=C(OCCN[C@@H](C)CC2=CC=C(OC)C(=C2)S(N)(=O)=O)C=CC=C1 | 0 | Train |
| 502 | CN(CCOC1=CC=C(NS(C)(=O)=O)C=C1)CCC1=CC=C(NS(C)(=O)=O)C=C1 | 0 | Train |
| 503 | CC(C)CN1C=NC2=C1C1=CC=CC=C1N=C2N | 0 | Train |
| 504 | CCOC1=CC=C(C[C@H]2NC(=O)CCSSC[C@H](NC(=O)[C@H](CC(N)=O)NC(=O)C(NC(=O)[C@@H](NC2=O)[C@@H](C)CC)[C@@H](C)O)C(=O)N2CCC[C@H]2C(=O)N[C@@H](CCCN)C(=O)NCC(N)=O)C=C1 | 0 | Train |
| 505 | CC[C@H](C)C(=O)O[C@H]1C[C@H](O)C=C2C=C[C@H](C)[C@H](CC[C@@H](O)C[C@@H](O)CC(O)=O)[C@@H]12 | 1 | Train |
| 506 | COC1=C(C)C2=C(C(=O)OC2)C(O)=C1C\C=C(/C)CCC(=O)OCCN1CCOCC1 | 1 | Train |
| 507 | CC(C)(OC1=CC=C(C=C1)C(=O)C1=CC=C(Cl)C=C1)C(O)=O | 1 | Train |
| 508 | C[C@]12CC[C@H]3[C@@H](CCC4=CC(=O)CC[C@]34C)[C@@H]1CC[C@@H]2O | 1 | Train |
| 509 | O=C1NC=NC2=C1C=NN2 | 1 | Test |
| 510 | [NH-]C1CCCCC1[NH-] | 1 | Train |
| 511 | CC1=CC=C(C=N1)C1=NC=C(Cl)C=C1C1=CC=C(C=C1)S(C)(=O)=O | 1 | Train |
| 512 | ClC1=C(NC2=NCCN2)C2=NSN=C2C=C1 | 1 | Train |
| 513 | N[C@@H](CCC(N)=O)C(O)=O | 1 | Train |
| 514 | CCC1NC(=O)C(C(O)C(C)C\C=C\C)N(C)C(=O)C(C(C)C)N(C)C(=O)C(CC(C)C)N(C)C(=O)C(CC(C)C)N(C)C(=O)C(C)NC(=O)C(C)NC(=O)C(CC(C)C)N(C)C(=O)C(NC(=O)C(CC(C)C)N(C)C(=O)CN(C)C1=O)C(C)C | 1 | Train |
| 515 | CCC1=C2NC3=C(CCOC3(CC)CC(O)=O)C2=CC=C1 | 1 | Test |
| 516 | COC1=C(OC)C=C2C(N)=NC(=NC2=C1)N1CCN(CC1)C(=O)C1COC2=C(O1)C=CC=C2 | 1 | Train |
| 517 | CCC(C)(C)C1=CC=C(CC(C)CN2CC(C)OC(C)C2)C=C1 | 0 | Test |
| 518 | CC1(C)OC2CC3C4CC(F)C5=CC(=O)C=CC5(C)C4(F)C(O)CC3(C)C2(O1)C(=O)CO | 0 | Train |
| 519 | CC1CC2C3CCC4=CC(=O)C=CC4(C)[C@@]3(F)C(O)CC2(C)[C@@]1(O)C(=O)CCl | 0 | Train |
| 520 | CCC1=CC=C(CCOC2=CC=C(CC3SC(=O)NC3=O)C=C2)N=C1 | 0 | Train |
| 521 | COC1=NC(C)=NC(Cl)=C1NC1=NCCN1 | 0 | Train |
| 522 | CCOC(=O)OC1(CCC2C3CCC4=CC(=O)C=CC4(C)C3C(O)CC12C)C(=O)COC(=O)CC | 0 | Test |
| 523 | CN1C2=C(C3=CC=CC=C13)C(=O)C(CN1C=CN=C1C)CC2 | 0 | Train |
| 524 | CN1CC(C=C2C1CC1=CNC3=CC=CC2=C13)C(=O)NC1(C)OC2(O)C3CCCN3C(=O)C(CC3=CC=CC=C3)N2C1=O | 0 | Test |
| 525 | CCC12CCC3C(CCC4=CC(=O)CCC34)C1CCC2(O)C#C | 0 | Test |
| 526 | CC1(O)CCC2C3CCC4=CC(=O)CCC4(C)C3CCC12C | 0 | Test |
| 527 | CCN(CC)C(=O)NC1CN(C)C2CC3=CNC4=CC=CC(=C34)C2=C1 | 0 | Train |
| 528 | CC12CC(=O)C3C(CCC4=CC(=O)C=CC34C)C1CCC2(O)C(=O)CO | 0 | Train |
| 529 | COC(C(OC1=NC(C)=CC(C)=N1)C(O)=O)(C1=CC=CC=C1)C1=CC=CC=C1 | 0 | Train |
| 530 | CCC(CO)NC(=O)C1CN(C)C2CC3=CNC4=CC=CC(=C34)C2=C1 | 0 | Train |
| 531 | CN(CC=CC1=CC=CC=C1)CC1=C2C=CC=CC2=CC=C1 | 0 | Train |
| 532 | [O-][N+](=O)OC1COC2C(COC12)O[N+]([O-])=O | 0 | Train |
| 533 | CC(C)C1CCC(CC1)C(=O)NC(CC1=CC=CC=C1)C(O)=O | 0 | Train |
| 534 | OC1=CC=C(O)C=C1 | 0 | Train |
| 535 | COC1=CC=C(C=C1)C1SC2=C(C=CC=C2)N(CC[NH+](C)C)C(=O)C1OC(C)=O | 0 | Train |
| 536 | ClC1=CC=C(COC(CN2C=CN=C2)C2=CC=C(Cl)C=C2Cl)C=C1 | 0 | Train |
| 537 | CC1=C(OC2=C(C=CC=C2C(=O)OCCN2CCCCC2)C1=O)C1=CC=CC=C1 | 0 | Train |
| 538 | CC1=CC(=O)N(O)C(=C1)C1CCCCC1 | 0 | Train |
| 539 | COC1=C2OC=CC2=CC2=C1OC(=O)C=C2 | 0 | Train |
| 540 | C[N+]1(C)C2CC(CC1C1OC21)OC(=O)C(CO)C1=CC=CC=C1 | 0 | Train |
| 541 | C[C@]12CC(=O)[C@H]3[C@@H](CCC4=CC(=O)CC[C@]34C)[C@@H]1CC[C@]2(O)C(=O)CO | 0 | Train |
| 542 | COC1=CC=C(CC2=C3C=C(OC)C(OC)=CC3=CC=N2)C=C1OC | 0 | Train |
| 543 | CCCCC(C)(O)CC=C[C@H]1[C@H](O)CC(=O)[C@@H]1CCCCCCC(=O)OC | 0 | Train |
| 544 | CC1(C)O[C@@H]2C[C@H]3C4CCC5=CC(=O)C=CC5(C)[C@H]4C(O)CC3(C)[C@@]2(O1)C(=O)CO | 0 | Train |
| 545 | CC12CCC3C(CCC4=C3C=CC(O)=C4)C1CCC2=O | 0 | Train |
| 546 | CCC12CC(=C)C3C(CCC4=CC(=O)CCC34)C1CC[C@@]2(O)C#C | 0 | Train |
| 547 | NC(CO)(CO)CO | 0 | Train |
| 548 | OC(=O)CCCCCCCC(O)=O | 0 | Train |
| 549 | C[N+]1(C)CCC(C1)OC(=O)C(O)(C1CCCC1)C1=CC=CC=C1 | 0 | Train |
| 550 | CC1(C)SC2C(NC(=O)C(C([O-])=O)C3=CC=CC=C3)C(=O)N2C1C([O-])=O | 0 | Test |
| 551 | CCCC1CC(N(C)C1)C(=O)NC(C(C)O)C1OC(SC)C(O)C(O)C1O | 0 | Train |
| 552 | NC1=CC=C(C=C1)S(N)(=O)=O | 0 | Test |
| 553 | NC(=O)C(C1CCN(CCC2=CC=C3OCCC3=C2)C1)(C1=CC=CC=C1)C1=CC=CC=C1 | 0 | Test |
| 554 | OC[C@H]1O[C@@](CO)(O[C@H]2O[C@H](CO)[C@@H](O)[C@H](O)[C@H]2O)[C@@H](O)[C@@H]1O | 0 | Train |
| 555 | NC(CCC(N)=O)C(O)=O | 0 | Train |
| 556 | CC1OC(CC(O)C1O)OC1C(O)CC(OC2C(O)CC(OC3CCC4(C)C(CCC5C4CC(O)C4(C)C(CCC54O)C4=CC(=O)OC4)C3)OC2C)OC1C | 0 | Train |
| 557 | CC1C(NC(=O)C(=NOC(C)(C)C(O)=O)C2=CSC(N)=N2)C(=O)N1S(O)(=O)=O | 0 | Train |
| 558 | CC(C)(C)NCC(O)COC1=NSN=C1N1CCOCC1 | 0 | Train |
| 559 | CC12CCC3C(CCC4=C3C=CC(O)=C4)C1CC(O)C2O | 0 | Test |
| 560 | CC(=O)[C@@]1(O)CC[C@H]2[C@@H]3C=C(C)C4=CC(=O)CC[C@]4(C)[C@H]3CC[C@]12C | 0 | Train |
| 561 | CC(=O)OCC(=O)C12OC3(CCCC3)OC1CC1C3CCC4=CC(=O)C=CC4(C)C3(F)C(O)CC21C | 0 | Train |
| 562 | C[C@@H]1CC[C@@]23CCC(=O)[C@H]2[C@]1(C)[C@@H](C[C@@](C)(C=C)[C@@H](O)[C@@H]3C)OC(=O)CSC1C[C@@H]2CC[C@H](C1)N2C | 0 | Train |
| 563 | CCN(CC)CC#CCOC(=O)C(O)(C1CCCCC1)C1=CC=CC=C1 | 0 | Train |
| 564 | NCC(O)=O | 0 | Train |
| 565 | CCOC(=O)N1C=CN(C)C1=S | 0 | Train |

**Table S2.** A comparison of the original RFC model with the top-performing models obtained from ten different random stratified splits.

| **Split** | **Accuracy** | **Precision** | **Recall** | **F1** | **Best Hyperparameters** |
| --- | --- | --- | --- | --- | --- |
| **Original** | **0.841** | **0.830** | **0.830** | **0.830** | **'n_estimators': 60, 'max_depth': 24, 'min_samples_split': 13, 'min_samples_leaf': 2** |
| **1** | 0.673 | 0.647 | 0.772 | 0.704 | 'n_estimators': 68, 'max_depth': 28, 'min_samples_split': 19, 'min_samples_leaf': 7 |
| **2** | 0.752 | 0.796 | 0.684 | 0.736 | 'n_estimators': 84, 'max_depth': 31, 'min_samples_split': 2, 'min_samples_leaf': 3 |
| **3** | 0.788 | 0.789 | 0.789 | 0.789 | 'n_estimators': 74, 'max_depth': 13, 'min_samples_split': 5, 'min_samples_leaf': 1 |
| **4** | 0.788 | 0.824 | 0.737 | 0.778 | 'n_estimators': 66, 'max_depth': 29, 'min_samples_split': 6, 'min_samples_leaf': 6 |
| **5** | 0.743 | 0.741 | 0.754 | 0.748 | 'n_estimators': 28, 'max_depth': 19, 'min_samples_split': 10, 'min_samples_leaf': 3 |
| **6** | 0.796 | 0.783 | 0.825 | 0.803 | 'n_estimators': 103, 'max_depth': 17, 'min_samples_split': 4, 'min_samples_leaf': 3 |
| **7** | 0.761 | 0.750 | 0.789 | 0.769 | 'n_estimators': 93, 'max_depth': 16, 'min_samples_split': 17, 'min_samples_leaf': 2 |
| **8** | 0.717 | 0.692 | 0.789 | 0.738 | 'n_estimators': 46, 'max_depth': 15, 'min_samples_split': 2, 'min_samples_leaf': 3 |
| **9** | 0.726 | 0.724 | 0.737 | 0.730 | 'n_estimators': 36, 'max_depth': 16, 'min_samples_split': 4, 'min_samples_leaf': 5 |
| **10** | 0.752 | 0.738 | 0.789 | 0.763 | 'n_estimators': 101, 'max_depth': 21, 'min_samples_split': 18, 'min_samples_leaf': 6 |

**Table S3.** Detailed description of RASAR descriptors.

| **RAfunction** | **Read-Across-based prediction function** |
| --- | --- |
| SD_activity | Variability in the response values for chosen similar source compounds corresponding to each target compound. |
| CV_activity | Relative variability of the response values for chosen nearby source compounds for every inquiry compound. |
| SE | Standard uncertainty in the observed response values for the chosen proximate source compounds related to each reference compound. |
| CV_similarity | Coefficient of variation of the similarity values |
| Avg. Sim | Mean similarity to the close source compounds for each query compound |
| Pos.Avg.Sim | Mean likeness of the proximate training compounds with observed activity values exceeding the average response of the training set. |
| Neg.Avg.Sim | Average similarity of the close training compounds having a lower observed activity value than the threshold (training set response mean) |
| SD_similarity | Variability in the likeness scores of the chosen nearby source compounds for every target compound. |
| MaxPos | Maximum Similarity level to the Positive close source compounds (based on source set observed response mean) |
| MaxNeg | Maximum Similarity level to the Negative close source set compounds |
| Abs MaxPos- MaxNeg | Absolute difference between MaxPos and MaxNeg |
| gm | Banerjee-Roy Coefficient |

**Table S4.** Summary of the classification-based qRASAR models.

| **Types** | **Training Set Metrics** | **Test Set Metrics** | **Figures** |
| --- | --- | --- | --- |
| EUC | Accuracy: 0.9956  Precision: 1.0000  Recall: 0.9915  F1 Score: 0.9957 | Accuracy: 0.7699  Precision: 0.7872  Recall: 0.6981  F1 Score: 0.7400 | 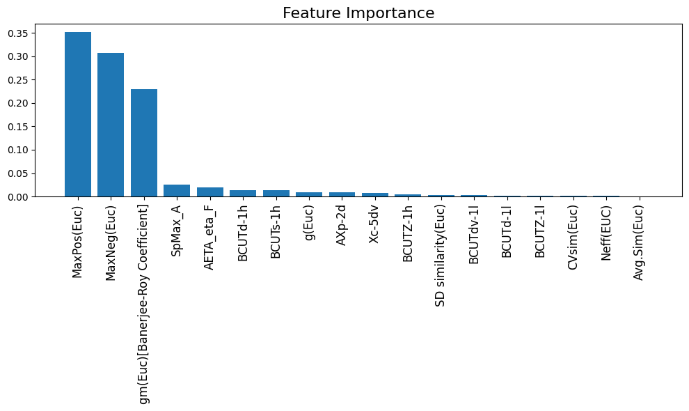 |
| EUC (Selected) | Accuracy: 0.9956  Precision: 0.9957  Recall: 0.9957  F1 Score: 0.9957 | Accuracy: 0.7568  Precision: 0.7400  Recall: 0.7255  F1 Score: 0.7327 | 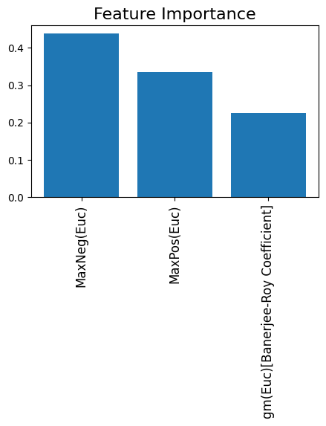 |
| GK | Accuracy: 0.9956  Precision: 0.9915  Recall: 1.0000  F1 Score: 0.9957 | Accuracy: 0.7434  Precision: 0.7222  Recall: 0.7358  F1 Score: 0.7290 | 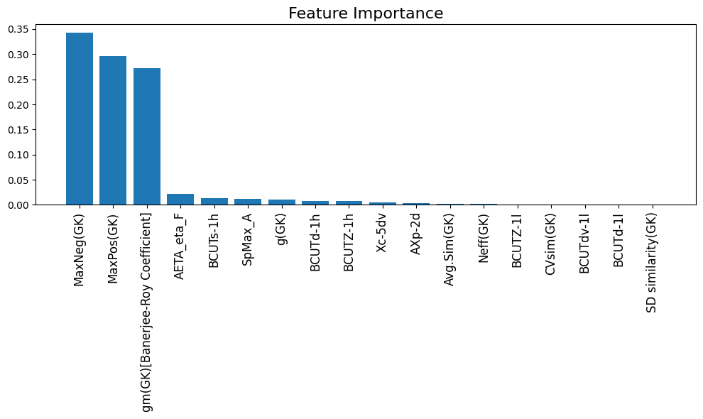 |
| GK (Selected) | Accuracy: 0.9956  Precision: 0.9915  Recall: 1.0000  F1 Score: 0.9957 | Accuracy: 0.7168  Precision: 0.6780  Recall: 0.7547  F1 Score: 0.7143 | 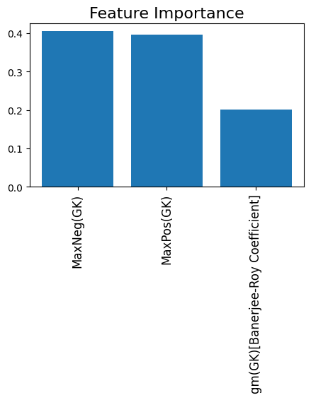 |
| LK | Accuracy: 0.9956  Precision: 0.9915  Recall: 1.0000  F1 Score: 0.9957 | Accuracy: 0.7788  Precision: 0.8043  Recall: 0.6981  F1 Score: 0.7475 | 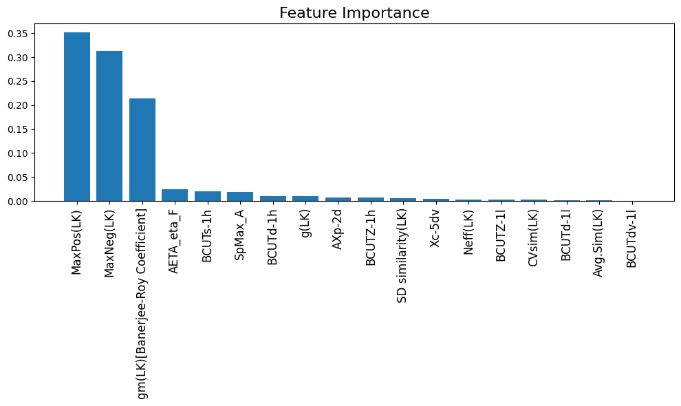 |
| LK (Selected) | Accuracy: 0.9956  Precision: 0.9915  Recall: 1.0000  F1 Score: 0.9957 | Accuracy: 0.6903  Precision: 0.6875  Recall: 0.6226  F1 Score: 0.6535 | 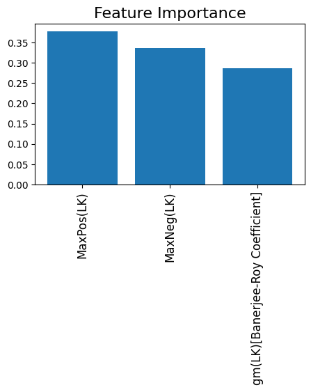 |

**Table S5**. Values of the descriptors of the outlier compounds.

| **ID** | ***AXp-2d*** | ***BCUTdv-1l*** | ***BCUTZ-1h*** | ***SpMax_A*** | ***BCUTd-1h*** | ***AETA_eta_F*** | ***BCUTZ-1l*** | ***Xc-5dv*** | ***BCUTs-1h*** | ***BCUTd-1l*** |
| --- | --- | --- | --- | --- | --- | --- | --- | --- | --- | --- |
| **119** | 0.577 | 0.996 | 8.029 | 1.732 | 3.034 | 0.451 | 5.876 | 0.000 | 8.002 | 0.968 |
| **171** | 0.500 | 0.547 | 16.001 | 1.618 | 2.111 | 0.038 | 5.894 | 0.000 | 4.005 | 0.986 |
| **200** | 0.260 | 1.968 | 7.053 | 2.525 | 3.231 | 0.676 | 5.683 | 0.128 | 3.046 | 1.751 |
| **352** | 0.315 | 0.989 | 8.024 | 2.325 | 3.141 | 0.962 | 4.982 | 0.058 | 7.009 | 0.977 |


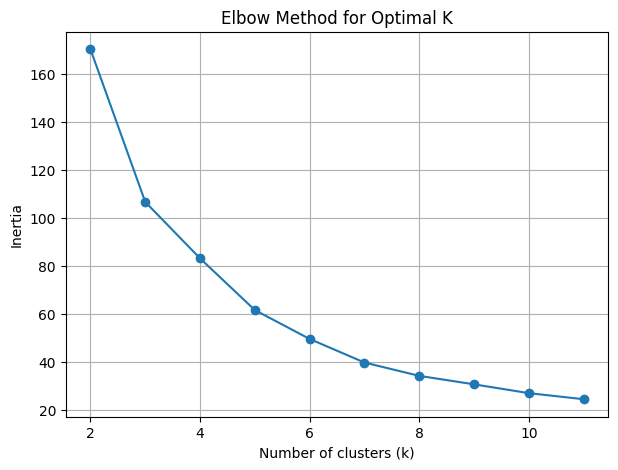


**Figure S1.** Elbow plot highlighted the relationship between the number of clusters (k) and the within-cluster sum of squares (WCSS). Here, the curve exhibits a noticeable “elbow” at k = 5, indicating that additional clusters beyond this point provide diminishing improvements in clustering performance. Therefore, five clusters were selected.

**
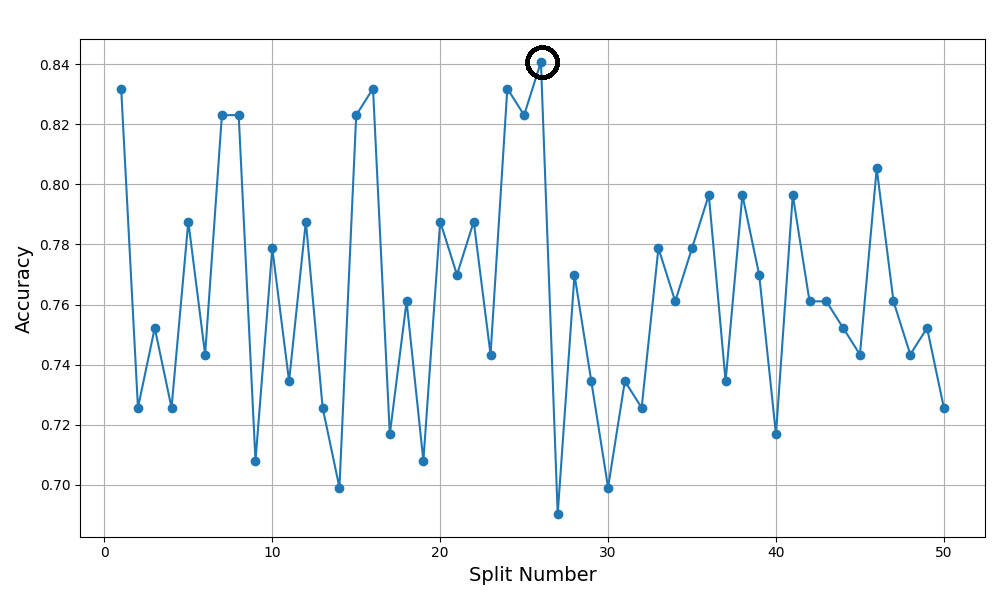
**

**Figure S2.** Accuracy across 50 independent stratified train-test splits (80:20). The highest-accuracy split (encircled black) was selected for downstream feature selection and model development.


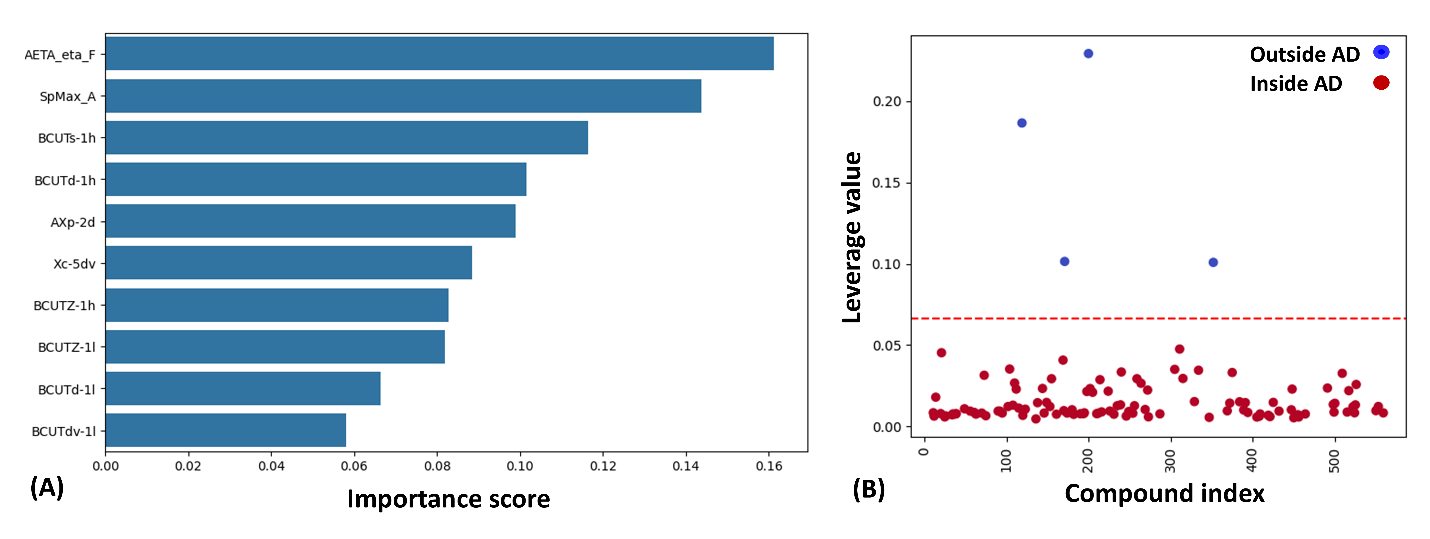


**Figure S3.** (A) The plot of selected descriptors with their importance score. (B) The plot of applicability domain (AD) based on the leverage approach. The outliers (those outside AD) identified by leverage highlighted in blue circles.
